# Exploring valid reference genes for gene expression studies in *Brachypodium distachyon *by real-time PCR

**DOI:** 10.1186/1471-2229-8-112

**Published:** 2008-11-07

**Authors:** Shin-Young Hong, Pil Joon Seo, Moon-Sik Yang, Fengning Xiang, Chung-Mo Park

**Affiliations:** 1 Department of Chemistry, Seoul National University, Seoul, 151-742, Korea; 2 Division of Biological Sciences and the Research Institute for Bioactive Materials, Chonbuk National University, Jeonju, 561-756, Korea; 3 School of Life Sciences, Shandong University, Jinan 250100, Shandong, PR China

## Abstract

**Background:**

The wild grass species *Brachypodium distachyon *(Brachypodium hereafter) is emerging as a new model system for grass crop genomics research and biofuel grass biology. A draft nuclear genome sequence is expected to be publicly available in the near future; an explosion of gene expression studies will undoubtedly follow. Therefore, stable reference genes are necessary to normalize the gene expression data.

**Results:**

A systematic exploration of suitable reference genes in Brachypodium is presented here. Nine reference gene candidates were chosen, and their gene sequences were obtained from the Brachypodium expressed sequence tag (EST) databases. Their expression levels were examined by quantitative real-time PCR (qRT-PCR) using 21 different Brachypodium plant samples, including those from different plant tissues and grown under various growth conditions. Effects of plant growth hormones were also visualized in the assays. The expression stability of the candidate genes was evaluated using two analysis software packages, geNorm and NormFinder. In conclusion, the ubiquitin-conjugating enzyme 18 gene (*UBC18*) was validated as a suitable reference gene across all the plant samples examined. While the expression of the polyubiquitin genes (*Ubi4 *and *Ubi10*) was most stable in different plant tissues and growth hormone-treated plant samples, the expression of the S-adenosylmethionine decarboxylase gene (*SamDC*) ranked was most stable in plants grown under various environmental stresses.

**Conclusion:**

This study identified the reference genes that are most suitable for normalizing the gene expression data in Brachypodium. These reference genes will be particularly useful when stress-responsive genes are analyzed in order to produce transgenic plants that exhibit enhanced stress resistance.

## Background

Quantification of gene expression levels is a fundamental step in virtually all aspects of molecular biological research. It is particularly important when genes that are expressed specifically under certain growth conditions are to be compared. Common experimental techniques used to evaluate gene expression levels include Northern blot hybridization and reverse transcription (RT)-mediated PCR (RT-PCR); both techniques are practical for analyzing expression of a small set of genes and are complemented by microarray analysis, which is routinely employed for large-scale, global expression profilings. In recent years, the qRT-PCR has been the method of choice for measuring gene expression levels in multiple samples involving a limited number of genes. It provides accurate and sensitive quantification of gene transcript levels, even for those genes with fairly low transcript levels are [1-3].

The popularity of the qRT-PCR method reflects the need for ways to quantitatively analyze gene expression data in the fields of molecular medicine, biotechnology, microbiology, and molecular diagnostics [4] as well as to accurately quantify mRNA levels in plants [5]. However, to avoid experimental deviations or the errors that inevitably occur during sample preparation procedures and data analysis, all of which make quantitation of gene transcripts unreliable, normalization of the qRT-PCR data is essential. The most common way to normalize the data is to use appropriate internal reference genes. An ideal reference gene is expressed at a constant level in all plant tissues under various growth conditions. Its expression should not be influenced by environmental changes or by exogenous application of growth hormones.

Commonly used reference genes in plant molecular biology, frequently called housekeeping genes, play a general role in basic cellular processes, such as cell structure maintenance and primary cellular metabolism, and thus are usually unaffected by external factors. For example, genes encoding tubulins, actins, or elongation factors are widely used in gene expression studies in *Arabidopsis thaliana *(Arabidopsis hereafter) and *Oryza sativa *(rice hereafter).

Brachypodium is a temperate wild grass species. Its morphological, genomic, and molecular genetic characteristics and simple growth requirements make it an ideal model system for grass biology [6]. Its genome size (approximately 355 Mbp) is smaller than that of rice. It also has a short growth cycle. A single plant life cycle can be completed within 6–8 weeks. Self-fertility, the availability of several diploid accessions, and its intrinsic resistance to diverse biotic and abiotic stress conditions are additional attributes.

Arabidopsis is widely used as a model system for all flowering plants. Rice has been used as a model for genomics research for all temperate grass species, including major cereals, such as barley and wheat. However, recent genomic analyses have revealed that the Brachypodium genome is phylogenetically closer to the genomes of economically important crops, such as wheat and barley, and several potential biofuel grasses, including switch grasses [7]. Brachypodium exhibits many agricultural traits similar to these grass crops. Furthermore, a protocol has recently been established for infecting Brachypodium with *Magnaporthe grisea *(rice blast) in order to explore its potential as a model system in which to study host-pathogen interactions [8]. To date, such molecular assays have routinely been carried out using rice or related monocots.

The rapidly growing interest in Brachypodium triggered the establishment of a series of genomic resources; these include the collecting of nuclear genomic sequences and bacterial artificial chromosome (BAC)/EST-based libraries in addition to the characterization of individual genetic resources [9]. Furthermore, the Brachypodium genome project is currently underway, and the full genomic sequence will be released to the public in the near future [7]. Several groups have also reported the existence of efficient *Agrobacterium*-mediated Brachypodium transformation systems [10,11], which are essential for functional genomics studies in Brachypodium. Such technological progress in Brachypodium research can help realize its potential as a new model system for biofuel grass biology, provided that stable reference genes are identified for gene expression studies in this plant species.

In this study, we report on reference genes that can potentially be used to normalize the results of gene expression studies in Brachypodium. Nine candidate genes, including those encoding actin (ACT7), elongation factor 1-alpha (EF1α, glyceraldehyde 3-phosphate dehydrogenase (GAPDH), rubisco activase (RCA), SamDC, alpha-tubulin (TUA6), UBC18, Ubi4, and Ubi10, were chosen, and partial gene sequences were extracted from the available Brachypodium databases . Brachypodium was either grown under various stress conditions or treated with an array of growth hormones. Different plant tissues and plants at different developmental stages were also included in the assays. Our data showed that while expression of the *UBC18 *gene was the most stable among all the plant samples examined, the *Ubi4 *and *Ubi10 *genes would be appropriate for analysis of gene expression studies in different plant tissues. Their expressions were also stable under the growth hormone treatments. In the meantime, the *SamDC *gene showed the most stable expression patterns in plants grown under diverse abiotic stress conditions. This work will certainly facilitate our future work on gene expression studies in biofuel grass crops as well as in Brachypodium.

## Results

### Selection of potential reference genes in Brachypodium

A handful of reference genes, including those encoding tubulins, actins, and elongation factors, are regularly used to normalize the RT-PCR or qRT-PCR data in Arabidopsis and rice. Additional genes, such as *elongation initiation factor 4a *(*ELF4a*), are sometimes employed to further standardize the data on plant samples grown under specific growth conditions. However, because none of the reference genes currently used in Arabidopsis and rice provides a stable expression under all the examined conditions, multiple reference genes must be included to ensure the normalization of the data in some molecular analyses.

Although genomic, cellular, and morphological aspects of Brachypodium have been widely explored in recent years, little information is available about molecular genetic analyses, and thus no systematic efforts have been made to identify its stable reference genes. Extensive literature searches have revealed that genes encoding various types of proteins, including enzymes and structural proteins, are widely used as internal controls for studies of plant gene expression through RT-PCR, qRT-PCR, and gene chip assays [12,13]. Analyzing these reference genes in other plant species led us to choose nine candidate reference genes in Brachypodium; *ACT7*, *RCA*, *EF1α*, *GAPDH*, *SamDC*, *TUA6*, *UBC18*, *Ubi4*, and *Ubi10*. Using the sequence information deposited in the HarvEST  under the GenBank accession numbers listed in Table 1, a series of qRT-PCR primers was designed which the sizes of the PCR products were approximately 100 – 300 bp (see Additional file 1).

**Table 1:** Selected reference genes used for gene expression studies in Brachypodium

**Gene symbol**	**Gene name**	**Brachypodium**	**Rice**	**Arabidopsis**
*ACT7*	Actin 7	DV478555	LOC_Os10g36650	AT5G09810
*EF1α*	Elongation factor 1-alpha	DV482887	LOC_Os03g08050	AT5G60390
*GAPDH*	Glyceraldehyde-3-phosphate dehydrogenase	DV482924	LOC_Os08g03290	AT3G04120
*RCA*	Rubisco activase	DV482669	LOC_Os11g47970	AT2G39730
*SamDC*	S-adenosyl methionine decarboxylase	DV482676	LOC_Os04g42090	AT3G25570
*TUA6*	Tubulin alpha-6	DV478602	LOC_Os11g14220	AT4G14960
*UBC18*	Ubiquitin-conjugating enzyme 18	DV481689	LOC_Os12g44000	AT5G42990
*Ubi4*	Polyubiquitin (Ubi4)	DV482834	LOC_Os04g53620	AT5G20620
*Ubi10*	Polyubiquitin (Ubi10)	DV484269	LOC_Os06g46770	AT4G05320

The Brachypodium gene accession numbers refer to the dbEST division of GenBank [25]. The Arabidopsis Genome Initiative (AGI) codes and the TIGR rice genome locus identifiers of the gene homologues are also listed.

### RNA integrity and amplification specificity

For qRT-PCR analyses, we included 21 different total RNA samples extracted from various plant materials. The plant materials analyzed included 5 different plant tissues (callus, leaf, root, spike, stem), plants at 4 different developmental stages (early vegetative, late vegetative, transition, and reproductive phases), and plants exposed to 4 different abiotic stress conditions, such as high salinity (300 mM NaCl), cold (4°C for 5 h), heat (42°C for 2 h), and drought (400 mM mannitol). Brachypodium seedlings were also treated with 8 different growth hormones; indole-3-acetic acid (IAA, 50 μM), brassinolide (BL, 50 μM), zeatin (50 μM), abscisic acid (ABA, 100 μM), gibberellic acid (GA, 50 μM), 1-aminocyclopropane-1-carboxylic acid (ACC, 50 μM), salicylic acid (SA, 100 μM), and methyl jasmonic acid (MeJA, 100 μM).

Prior to carrying out qRT-PCR reactions, the viability of all RNA samples was examined using RT-PCR to evaluate the expression of the *GAPDH *gene. All the RT-PCR reactions produced a single *GAPDH*-specific band with a predicted molecular size (approximately 500 bp) on a 1.2% agarose gel visualized with ethidium bromide staining (see Additional file 2), confirming the hypothesis that the RNA samples extracted from Brachypodium plant materials are appropriate for transcript level analysis. The integrity and quality of RNA samples were further evaluated by electrophoretic analysis using the Labwork Image Acquisition and Analysis software (Media Cybernetics) (see Additional file 2).

The results showed that the *GAPDH *gene was stably expressed under various abiotic stress conditions and not discernibly affected by diverse growth hormones, although it exhibited less stability in some plant tissues (see Additional file 2).

To examine the stability of reference gene expressions, transcript levels were measured by qRT-PCR. Aliquots of the primary cDNA synthesis reaction mixture were used in qRT-PCR to amplify gene-specific primer pairs of each of the nine candidate reference genes. Primer dimer formation and nonspecific amplification can falsely increase gene transcript levels and thus must be avoided, especially when qRT-PCR is carried out using the SYBR green dyes. Gene-specific amplification of each of the nine candidate genes was confirmed by the appearance of a single, dominant peak in the qRT-PCR dissociation curve analyses (see Additional files 3, 4, 5, 6, 7, 8, 9, 10, 11).

### Identification of marker genes for growth hormone treatments

A set of marker genes that are regulated specifically by growth hormones and environmental factors is a prerequisite for determining reference gene expression stability under various experimental conditions. Since there is no information on marker genes available in Brachypodium, we screened potential marker genes in the Brachypodium databases that are homologous to those well characterized in Arabidopsis.

Transcript levels of the selected marker genes were compared by qRT-PCR in Brachypodium plants treated either with various growth hormones or with abiotic stresses (see Additional file 12). We found that Brachypodium genes, which are homologous to *IAA1*, *ARR4*, *BAS1*, *NPR1*, *CBF3*, and *HSC70*, were significantly induced by the relevant treatments. In contrast, genes that are homologous to *GA3OX2-1*, *RD22*, and *Chitinase1 *were repressed under the conditions examined (see Additional files 13 and 14). These observations indicate that treating Brachypodium seedlings with growth hormones or growing seedlings under abiotic stress conditions conferred regulatory effects on the selected marker genes similar to those found in Arabidopsis.

### Expression data analysis

**Figure 1:**
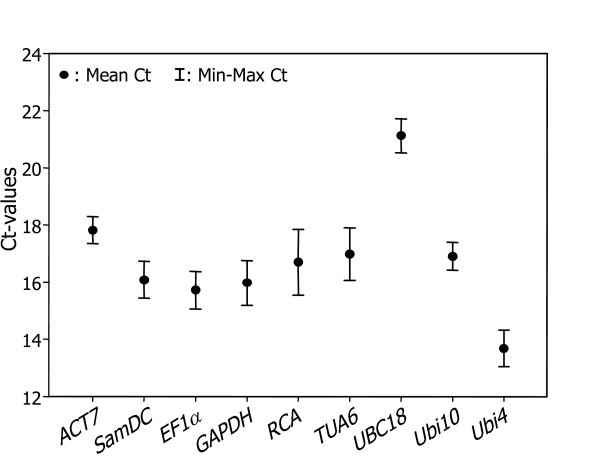
**Expression levels of candidate reference genes in different plant samples**. The scatter plots exhibit the expression levels of candidate reference genes in the tested Brachypodium samples (n = 21). Values are given as cycle threshold numbers (Ct values) with a mean of duplicate samples. Bars indicate standard error of the mean.

A simple, widely used way to identify stably expressed genes is to calculate cycle threshold (Ct) values in the qRT-PCR reactions. The Ct value represents the cycle at which a significant increase of the PCR product, which is measured by an increase in fluorescence, occurs; this cycle is generally marked by the middle of the exponential phase of amplification. Gene expression analyses of the nine reference genes exhibited a narrow mean Ct value range across all the experimental samples (Figure 1). The Ct values ranged from 13 to 22, while most of the values were distributed between 15 and 19. *Ubi4 *was the most abundantly transcribed, reaching the threshold fluorescence peak after 13 amplification cycles. The Ct average of all other genes was approximately 17 cycles. As a result, the *Ubi4 *transcript level was approximately 16 times more abundant than the average of the data set. The least abundant transcripts were those of *UBC18 *having a Ct value of 19 or higher. Most of the reference genes had relatively smaller variations in gene expression (below 1 cycle) except for *RCA*.

The geNorm software was employed as a means of determining the expression stability of the selected reference genes [14]. This program automatically calculates the average expression stability value (M) as the average pairwise variation (V) of a particular gene with all other control genes and determines the V values with all other control genes as the standard deviation of the logarithmically transformed expression ratios.

**Figure 2:**
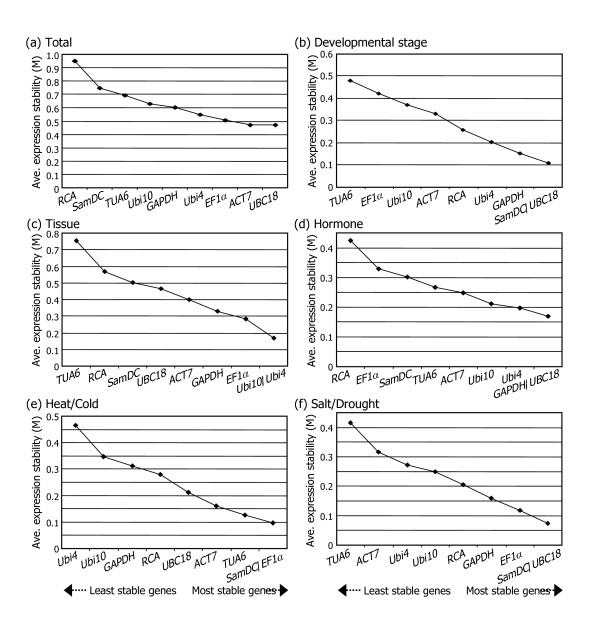
**Average expression stability values (M) of the candidate reference genes**. Average expression stability values (M) of the reference genes were measured during stepwise exclusion of the least stable reference genes. A lower M value indicates more stable expression, as analyzed by the geNorm software in plant samples at different developmental stages (b), plant tissue samples (c), hormone-treated samples (d), heat- and cold-treated samples (e), and in high salt- and drought-treated samples (f). The M values calculated for all plant samples examined are also given (a).

Figure 2 displays the M values of remaining reference genes at each step during stepwise exclusion of the least stable reference gene. Starting from the least stable gene on the left, the genes are ranked according to increasing expression stability, ending with the two most stable genes on the right. The most stable reference gene was not identical in individual plant samples. While the *SamDC *and *UBC18 *genes were most stably expressed throughout the plant life cycle (Figure 2b), the *Ubi4 *and *Ubi10 *genes ranked highest in different plant tissue samples with an M value of 0.169 (Figure 2c). For the growth hormone-treated samples, the most stable genes were *GAPDH *and *UBC18 *with an M value of 0.169 (Figure 2d). The *EF1α*, *UBC18*, and *SamDC *genes ranked high in Brachypodium seedlings grown under abiotic stress conditions, indicating that these genes are stably expressed and probably play a housekeeping role (Figures 2e and 2f). All the tested samples reached a high expression stability with relatively low M values of less than 1, which are far below the default limit of M ≤ 1.5 [14].

Examining all the expression data examined revealed that *UBC18 *and *ACT7 *are the most stable genes that might be widely used for multiple purposes (Figure 2a). In contrast, *RCA *was the least stable among the genes examined.

**Figure 3:**
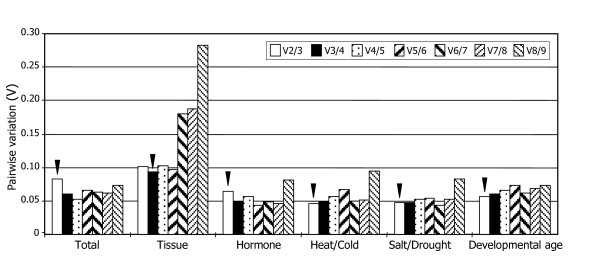
**Pairwise variation (V) analysis of the candidate reference genes**. The pairwise variation (V_n_/V_n+1_) was analyzed between the normalization factors NF_n _and NF_n+1 _by the geNorm software to determine the optimal number of reference genes required for qRT-PCR data normalization.

To determine the optimal number of reference genes required for accurate normalization, the geNorm software was used to calculate the pairwise variation (V_n_/V_n+1_) between the sequential normalization factors (NF) (NF_n _and NF_n+1_) (Figure 3). In the original publication describing geNorm [14], a threshold of 0.15 or lower for the pairwise variation was proposed to indicate that inclusion of an additional reference gene is unnecessary. However, 0.15 is not a strict cut-off value but an ideal value. Therefore, the graph displayed in Figure 3 is intended only to provide guidance for determining the optimal number of reference genes. In addition, the observed trend of changing V values when using additional genes can be equally informative. Therefore, in most cases, the use of three reference genes results in much more accurate and reliable normalization than only one gene and is thus considered to be a valid normalization strategy. We therefore decided to take the threshold of 0.1 as a cut-off value for the inclusion of our reference genes.

Analysis of the data obtained from different plant tissues using the geNorm software produced a plot displaying the average expression stability values of the candidate reference genes in each round of the analysis, ranking them from the least stable gene at the far left to the two most stable genes at the far right (Figure 2). Evaluation of different plant tissues revealed a significant decrease in the pairwise variation with the inclusion of a fourth gene (note the difference in the V values of V2/3 and V3/4 in Figure 3). The pairwise variation at the V3/4 value was 0.086, which is below the threshold of 0.1, indicating that the normalization factor should preferably contain at least three reference genes. Therefore, it would be ideal to include *Ubi4*, *Ubi10*, and *EF1α *as reference genes to normalize gene expression data in plant tissues.

Analysis of the pairwise variation in the growth hormone-treated samples revealed that a V score of 0.064 is achieved with two reference genes (Figures 2d and 3), indicating that the average of the top two reference genes, *GAPDH *and *UBC18*, is the optimal normalization factor for gene expression studies in the growth hormone-treated Brachypodium plant materials. In the heat- and cold-treated plant samples, *SamDC *and *EF1α *were the most stable reference genes (Figure 2e). As in the growth hormone-treated samples, the pairwise variation at V2/3 was below the threshold of 0.1, and thus two reference genes, *SamDC *and *EF1α*, would be sufficient (Figure 3).

In the meantime, evaluation of the salt- and drought-treated samples did not exhibit any discernible difference in the pairwise variation with the inclusion of a third gene (Figures 2f and 3). It is apparent that the average of the top two reference genes, *SamDC *and *UBC18*, is the optimal normalization factor for gene expression studies in the salt- and drought-treated samples.

To further confirm the results obtained by the geNorm program, we also employed the Normfinder software , an algorithm for identifying the optimal normalization gene among a set of candidate genes. This software ranks the set of candidate normalization genes according to the stability of their expression patterns in a given sample set analyzed in a given experimental design [15]. The results of the Normfinder analysis applied to our data sets are summarized in Table 2. The ranking of the reference genes was almost identical to that determined by the geNorm software. The *Ubi4 *and *Ubi10 *genes still ranked highest for different plant tissues and growth hormone-treated plant samples, while the *GAPDH *or *SamDC *genes appeared to be the highest reliable controls for plant samples exposed to abiotic stress.

**Table 2:** Brachypodium reference genes for normalization and their expression stability values calculated by the NormFinder software

**Rank**	**Tissue**		**Growth hormone**		**Heat/Cold**		**High salt/Drought**		**Developmental stage**		**Total**	
	Gene	Stability	Gene	Stability	Gene	Stability	Gene	Stability	Gene	Stability	Gene	Stability
1	*Ubi4*	0.0846	*Ubi10*	0.1377	*GAPDH*	0.1723	*SamDC*	0.0372	*Ubi4*	0.0987	*UBC18*	0.2586
2	*Ubi10*	0.0846	*Ubi4*	0.1640	*EF1α*	0.1951	*UBC18*	0.0378	*SamDC*	0.0996	*Ubi4*	0.3363
3	*GAPDH*	0.2751	*GAPDH*	0.1768	*SamDC*	0.2164	*GAPDH*	0.0738	*UBC18*	0.156	*EF1α*	0.3876
4	*EF1α*	0.2796	*ACT7*	0.2053	*ACT7*	0.2434	*EF1α*	0.1951	*GAPDH*	0.2276	*SamDC*	0.4791
5	*ACT7*	0.3708	*TUA6*	0.2103	*RCA*	0.2689	*RCA*	0.2063	*ACT7*	0.3401	*ACT7*	0.4845
6	*UBC18*	0.5032	*UBC18*	0.2291	*Ubi10*	0.3139	*Ubi10*	0.3426	*RCA*	0.3682	*TUA6*	0.5777
7	*SamDC*	0.5512	*EF1α*	0.3028	*TUA6*	0.3279	*ACT7*	0.3441	*Ubi10*	0.3776	*Ubi10*	0.5937
8	*RCA*	0.8238	*SamDC*	0.3946	*UBC18*	0.3309	*Ubi4*	0.4078	*EF1α*	0.6029	*GAPDH*	0.6658
9	*TUA6*	1.3712	*RCA*	0.7124	*Ubi4*	0.8483	*TUA6*	0.7401	*TUA6*	0.6579	*RCA*	0.926

In each column, a low stability value as an estimate of the combined intra- and intergroup variation of the respective gene represents a high gene expression stability of the respective gene between the matched samples.

## Discussion

The qRT-PCR is broadly accepted as the method of choice for accurate and sensitive quantification of gene transcript levels, even for those genes whose transcript levels are low. For valid qRT-PCR analysis, accurate normalization of gene expression against an appropriate internal control is required. Most gene expression studies in the literature generally use a single internal control for normalization [16], and the validity of the conclusion depends highly on the control applied. Therefore, it is necessary to validate the expression stability of the control gene under specific experimental conditions prior to its use for normalization.

The most commonly used reference genes in the pre-genomic era were chosen primarily for their known or suspected housekeeping roles in basic cellular processes. The actin gene, one such reference gene, is used in normalizing the quantification of gene expression levels [17]. Genes encoding GAPDH, actin, and EF1α have been used as the most relevant reference genes for berry development [18]. However, the actin gene was found to be inappropriate for use as a reference gene, since some variations of the transcript levels were observed in different plant tissues and plants grown under different growth conditions.

Our study showed that the polyubiquitin genes (*Ubi4 *and *Ubi10*) and the *UBC18 *gene encoding an ubiquitin-conjugating enzyme exhibited the most stable expression in different plant tissues or in plants treated with various growth hormones. These results are also supported by the previous observation in which *Ubi10 *showed a highly stable expression pattern in Arabidopsis [12]. An ubiquitin gene (*UBQ*) and a *TUA *gene are the most stably expressed in plant tissue samples from poplar [12]. Notably, the *UBQ *gene is the most stably expressed in the parasitic plant *Orobanche ramose *[19]. On the other hand, the expression of *UBQ5 *and *EF1α *was the most stable among all the plant tissues of rice [20]. In potato, *EF1α *is also the most stably expressed in plants grown under biotic and abiotic stress conditions [21]. In addition, *GAPDH *exhibited the most stable expression in different plant tissues of sugarcane [22]. Although genes encoding ubiquitins and polyubiquitins are stably expressed in different plant tissues in several plant species, these genes may not be stably expressed in other plant species. The reference genes appear to be regulated differentially in different plant species and cell types or under different experimental conditions. Therefore, no single reference gene with stable expression is suitable for normalizing all expression data obtained from various experimental conditions.

Our data demonstrate that *UBC18 *and *SamDC *are a reliable set of reference genes for normalizing gene expression data when multiple Brachypodium samples are analyzed (Figure 2). In particular, *UBC18 *showed acceptable expression stability in all the plant samples examined. Accumulation of the *SamDC *transcripts is ubiquitous in different plant organs in Brachypodium, including the callus, spike, root, leaf, and sheath. Therefore, it is not surprising that *SamDC *seems to be essential for embryo development in Arabidopsis [23]. It is likely that *GAPDH *and *SamDC *play a housekeeping role in Brachypodium growth and development.

While the geNorm software is used to identify not only the most stable gene but also the optimum pair of genes with least variation in their expression ratios, the NormFinder software is able to identify the gene with the most stable expression. Our results lead us to propose that the mean data rendered by *UBC18*, *SamDC*, *Ubi4*, and *Ubi10 *be used to normalize gene expression values in Brachypodium more accurately. To our knowledge, this is the first systematic attempt to identify reference genes in Brachypodium.

The Brachypodium genome project is currently underway under the auspices of the US Department of Energy. A draft genomic sequence is expected to be released in the near future, which will trigger diverse molecular genetic studies on Brachypodium as well as on related grass species. Of particular interest is how the plant responds to environmental stresses. However, there is no report so far on the stress-responsive genes or those involved in growth hormone signaling in Brachypodium. In this work, we searched for potential marker genes mediating stress responses and growth hormone signaling. Most of the selected marker genes exhibited responses to abiotic stresses and growth hormones that were similar to those of the Arabidopsis gene homologues, suggesting that the known stress-responsive genes and underlying molecular mechanisms in Arabidopsis are conserved in Brachypodium. One exception is the Brachypodium *RD22 *gene. While the Arabidopsis *RD22 *gene (*AtRD22*) is induced by ABA and abiotic stresses, the Brachypodium *RD22 *gene is repressed by ABA, high salt, and drought (see Additional files 13 and 14). At least some Brachypodium genes, such as *RD22*, likely play a role that is characterized by molecular mechanisms distinct from those exerted by the Arabidopsis gene homologues.

## Conclusion

We identified several reference genes that are suitable for qRT-PCR data normalization in Brachypodium. This is the first systematic exploration of valid reference genes in this plant species. Although most of the selected candidate genes exhibited stable expression patterns acceptable for reference genes, different genes showed the highest stability in different plant samples. While expression of *Ubi4 *and *Ubi10 *was most stable in different plant tissues and growth hormone-treated plant samples, that of *SamDC *was most stable under different environmental conditions. Overall, *UBC18 *is our reference gene of choice for analyzing multiple Brachypodium samples.

## Methods

### Plant sample preparation

A community standard diploid inbred line of *Brachypodium distachyon*, Bd21, was used for all experimental treatments. The palea and lemma were carefully peeled off from the mature seeds with fine forceps. The stripped seeds were sterilized by soaking in a solution of 10% household bleach (5.25% NaOCl) supplemented with 0.1% Tween 20 for 10 min with occasional rocking. The sterilized seeds were thoroughly rinsed three times with sterile double-distilled water and placed on Murashige and Skoog (MS)-agar plates (MS-agar plates hereafter) (4.3 g/l MS salts with vitamins, 3% sucrose, pH 5.8, and 0.4% phytagel). The seeds were cold-treated at 4°C for 2 days to synchronize germination and allowed to germinate in a culture room controlled at 25°C with a daily photoperiodic cycle of 16 h light and 8 h dark.

Four-week-old Brachypodium plants were used for treatments with growth hormones and abiotic stresses. Harvested plant samples were frozen in liquid nitrogen and stored at -80°C until RNA extraction. For gene expression studies in different plant tissues, appropriate plant tissues were harvested from 5-week-old, fully-grown plants. For gene expression studies at different developmental stages, plants were harvested at 7 days after germination (DAG) (early vegetative phase), 12 (late vegetative phase), 20 (transition phase), and 30 (reproductive phase) (see Additional file 2).

### Growth hormones and abiotic stresses

For growth hormone treatments, 4-week-old Brachypodium plants were transferred to the MS liquid cultures supplemented with IAA (50 μM), brassinolide (BL, 50 μM), zeatin (50 μM), ABA (100 μM), GA (50 μM), 1-aminocyclopropane-1-carboxylic acid (ACC, 50 μM), SA (100 μM), or with MeJA (100 μM) and incubated for 5 h with gentle shaking. The mock seedlings were similarly incubated in a MS liquid but without adding growth hormones.

For salt and drought stress treatments, 4-week-old Brachypodium plants were transferred to the MS liquid cultures supplemented either with 300 mM NaCl or with 400 mM mannitol, respectively, and gently shaken for 5 h. For cold and heat treatments, the seedlings were incubated at 4°C for 5 h or 42°C for 2 h, respectively.

### Selection of potential reference genes in Brachypodium

To identify potential Brachypodium homologues of the Arabidopsis or rice genes commonly used as internal controls for gene expression studies, we queried Version 0.52 of the HarvEST:Brachypodium software , which displays 6 different libraries from Brachypodium. All gene sequences were obtained from the GenBank dbEST database. The HarvEST:Brachypodium software contains best BLASTX hits from the UniProt and the rice and Arabidopsis genomes (TIGR version 5, February 2007, and TAIR version 7, April 2007, respectively).

The HarvEST is principally an EST database-viewing software that emphasizes gene function and is oriented to comparative genomics and design of oligonucleotides, with an aim to support diverse research activities, such as microarray content design, functional annotation, and physical and genetic mapping . The HarvEST:Brachypodium is the most recent and standardized EST database for Brachypodium and thus can be used to examine sequence alignments and determine where individual sequences reliably deviate from a consensus sequence.

Selected Brachypodium ESTs of potential reference genes were used to design primers. A set of qRT-PCR primers with high-efficiency for nine individual reference genes was designed using the Primer3 software (version 0.4.0) [24]. The primers were designed to have melting temperatures in a range of 50 – 60°C, depending on individual genes. Their sequences are summarized in Additional file 1.

### Total RNA extraction and primary cDNA synthesis

Total RNA was extracted from appropriate plant samples using the RNeasy Plant mini kit (Qiagen, Valencia, CA) according to the manufacturer's procedure. The quality and integrity of the RNA samples were evaluated by absorbance measurements and by electrophoretic analysis using the Labwork Image Acquisition and Analysis Program (Media Cybernetics, San Diego, CA). All the RNA samples used in the qRT-PCR reactions showed a 260/280 nm absorbance ratio of 1.8 – 2.2. RNA samples with a ratio of ≈ 2 are generally qualified for subsequent enzymatic reactions. Prior to RT-PCR and qRT-PCR, total RNA samples were pretreated with a RNase-free DNase I to eliminate any contaminating genomic DNA. The primary cDNA was synthesized from approximately 3 μg of total RNA using the MMLV first-strand synthesis system (Promega, Madison, WI) and the oilgo-dT and random primers in a reaction volume of 40 μl according to the manufacturer's procedure.

To rule out any genomic DNA contamination in the RNA preparations, the RNA samples and genomic DNA were subject in parallel to PCR amplifications of the *ARR4 *and *GAPDH *gene sequences (30 cycles), and the PCR products were compared. No visible amplifications of genomic DNAs were detected from the RNA samples (see Additional file 15).

### RT-PCR and qRT-PCR

One μl of the primary cDNA synthesis reaction mixture was taken for subsequent PCR amplification by RT-PCR or qRT-PCR. RT-PCR runs were routinely carried out for 20 to 35 cycles, depending on the linear range of PCR amplification for each gene. Each PCR cycle included incubations at 94°C for 30 s, at 55°C for 1 min, and at 72°C for 5 min. One additional cycle at 72°C for 10 min was run after the last cycle to allow trimming of incomplete polymerizations. Positive and negative control genes were included in the reaction sets to ensure the feasibility of the assay conditions. The RT-PCR primers used are listed in Additional file 1.

qRT-PCR reactions were carried out in 96-well blocks with an Applied Biosystems 7500 Real-Time PCR System using the SYBR Green I master mix in a reaction volume of 25 μl, which contains 1 μl of the primary cDNA reaction mixture, 2X SYBR Green PCR Master Mix (Applied Biosystems, Foster City, CA), and a primer pair. The primers used are listed in Additional file 1. The two-step thermal cycling profile used was 15 s at 95°C and 1 min at 60°C. All qRT-PCR reactions were carried out in biological duplicates, each of which was used for RNA extraction followed by qRT-PCR in triplicate. The final threshold cycle (C_t_) values were the mean of six values (biological duplicates, each with triplicate). The comparative ΔΔC_t _method was used to evaluate the relative quantities of each amplified product in the samples. The C_t _was automatically determined for each reaction by the Applied Biosystems 7500 Real-Time PCR System set with default parameters. The specificity of the qRT-PCR reactions was determined by melt curve analysis of the amplified products using the standard method installed in the System.

Each qRT-PCR reaction set included a negative control with water instead of cDNA. Duplicate measurements were averaged, and the mean values were used for further calculations.

### Determining expression stability of reference genes

The expression stability of each reference gene was analyzed using the geNorm (version 3.5) and NormFinder (version 0.953) software packages, which are also integrated into the GenEx software (version 4.3.5, ). The geNorm software calculates the gene expression stability (M) for a reference gene as the average pairwise variation V for the gene with all other tested reference genes. Stepwise exclusion of the gene with the highest M value allows the tested genes to be ranked according to the stability of their expression patterns.

The NormFinder software is an algorithm for identifying the optimal normalization gene among a set of candidate genes. It ranks the set of candidate normalization genes according to the stability of their expression patterns in a given sample set under a given experimental design. Therefore, it can analyze expression data obtained through any quantitative method, such as qRT-PCR and microarray-based expression profiling. The lowest stability value represents the most stable gene expression within the gene set examined.

## Abbreviations

ACT7: actin 7; Ct value: cycle threshold; EF1α: elongation factor 1-alpha; EST: expressed sequence tag; GAPDH: glyceraldehyde-3-phosphate dehydrogenase; qRT-PCR: quantitative real-time RT-PCR; RT-PCR: reverse transcription-PCR; RCA: rubisco activase; SamDC: S-adenosylmethionine decarboxylase proenzyme; TUA6: tubulin alpha-6; UBC18: ubiquitin-conjugating enzyme 18; Ubi10: Polyubiquitin (Ubi10); Ubi4: Polyubiquitin (Ubi4).

## Authors' contributions

SYH and PJS performed all the experimental procedures and statistical calculations jointly. FX established the culture conditions for Brachypodium and provided technical support. MSY provided technical assistance and scientific discussion. CMP conceived the project, supervised the study design, and contributed to writing the manuscript.

## Supplementary Material

Additional file 1 **PCR primers used in this work.** The primers were designed using the Primer3 software (version 0.4.0) [24]. They had melting temperatures in a range of 50 – 60°C, depending on individual genes. *The primer set was used only for RT-PCR analysis of *GAPDH*. All other primers except for this were used for qRT-PCR reactions. Click here for file

Additional file 2 **RT-PCR analyses of *GAPDH *transcript levels in different Brachypodium samples.** Total RNA samples were isolated from plants grown under abiotic stresses (a), from different plant tissues (b), from plants treated with various growth hormones (c), or from plants at different developmental stages (d). The quality of the RNA samples was determined by electrophoretic analysis using the Labwork Image Acquisition and Analysis Program (Media Cybernetics, see **Methods**). Click here for file

Additional file 3 **Dissociation curve data for *UBC18 *in growth hormone-treated samples.** Click here for file

Additional file 4 **Dissociation curve data for *Ubi4 *in growth hormone-treated samples.** Click here for file

Additional file 5 **Dissociation curve data for *Ubi10 *in growth hormone-treated samples.** Click here for file

Additional file 6 **Dissociation curve data for *RCA *in growth hormone-treated samples.** Click here for file

Additional file 7 **Dissociation curve data for *TUA6 *in growth hormone-treated samples.** Click here for file

Additional file 8 **Dissociation curve data for *GAPDH *in growth hormone-treated samples.** Click here for file

Additional file 9 **Dissociation curve data for *EF1α *in growth hormone-treated samples.** Click here for file

Additional file 10 **Dissociation curve data for *SamDC *in growth hormone-treated samples.** Click here for file

Additional file 11 **Dissociation curve data for *ACT7 *in growth hormone-treated samples.** Click here for file

Additional file 12 **Brachypodium marker genes for gene expression studies in response to growth hormone treatments and abiotic stresses.** The Brachypodium genes homologous to the Arabidopsis or rice genes that are regulated by growth hormones or abiotic stresses were examined by qRT-PCR. The sources of the gene accession numbers are those described in Table 1. Click here for file

Additional file 13 **qRT-PCR data on Brachypodium genes regulated by growth hormones and abiotic stresses.** Transcript levels were measured by qRT-PCR. Bars mark the standard error of the mean. The mean values were used as positive controls to determine the effects of growth hormone and abiotic stresses on reference genes. The gene accessions and the effects of each treatment on individual genes were summarized in Additional file 12. Click here for file

Additional file 14 Validation of the most suitable gene *UBC18 *using plants treated with ABA or high salt. RNA samples isolated from plants treated with ABA or high salt, as used in Additional file 13, were subject to RT-PCR of the *UBC18 *and *TUA *genes. The RT-PCR products were evaluated by electrophoretic analysis using the Labwork Image Acquisition and Analysis Program (Media Cybernetics). Click here for file

Additional file 15 **Genomic PCR and RT-PCR amplifications of the *ARR4 *and *GAPDH *genes.** (a) The *ARR4 *and *GAPDH *gene structures. The gene sequences were extracted from the JGI 4X Brachy Sequence produced by the US Department of Energy Joint Genome Institute . The gene structures were predicted using the GENSCAN server . The black boxes denote exons. The numbers in parentheses indicate the sizes of exons and introns in base pairs (bp). The arrowheads mark the position and direction of the PCR primers. (b) Genomic PCR and RT-PCR. The PCR reactions were carried out for 30 cycles using either genomic DNA (gDNA) or primary cDNA (cDNA) synthesized from total RNA pretreated with a RNase-free DNase I. The PCR primers were those listed in Additional file 1. The numbers in parentheses indicate the sizes of PCR products in bp. The arrow marks a predicted PCR product. SM, size marker. Click here for file
