# Supplementary material for: Exploring valid reference genes for gene expression studies in Brachypodium distachyon by real-time PCR
Source: BMC Plant Biol. 2008 Nov 7;8:112. doi: 10.1186/1471-2229-8-112 (PMC2588586; doi:10.1186/1471-2229-8-112)
Supplement: Additional file 1 — PCR primers used in this work. The primers were designed using the Primer3 software (version 0.4.0) [24]. They had melting temperatures in a range of 50 – 60°C, depending on individual genes. *The primer set was used only for RT-PCR analysis of GAPDH. All other primers except for this were used for qRT-PCR reactions. [file 1471-2229-8-112-S1.doc]

**Additional file 15: PCR primers used in this work**

| Gene  symbol | cDNA  /EST | GeneBank  number | Polarity | Amplicon  length | Primer sequence (base positions in the EST clones) |
| --- | --- | --- | --- | --- | --- |
| *ACT7* | EST | DV471671 | Forward | 188 bp | 5’-CCTGAAGTCCTTTTCCAGCC (339-359) |
|  |  |  | Reverse |  | 5’-AGGGCAGTGATCTCCTTGCT (526-508) |
| *EF1α* | EST | DV482887 | Forward | 184 bp | 5’-CCATCGATATTGCCTTGTGG (275-294) |
|  |  |  | Reverse |  | 5’-GTCTGGCCATCCTTGGAGAT (458-439) |
| *GAPDH* | EST | DV482924 | Forward | 236 bp | 5’-TTGCTCTCCAGAGCGATGAC (139-158) |
|  |  |  | Reverse |  | 5’-CTCCACGACATAATCGGCAC (374-355) |
| *RCA* | EST | DV482669 | Forward | 209 bp | 5’-ACCAGCTTCCTTGGGAAGAA (244-263) |
|  |  |  | Reverse |  | 5’-AGCGAGTCGACGATACCCTT (452-433) |
| *SamDC* | EST | DV482676 | Forward | 190 bp | 5’-TGCTAATCTGCTCCAATGGC (1678-1697) |
|  |  |  | Reverse |  | 5’-GACGCAGCTGACCACCTAGA (1867–1848) |
| *TUA6* | EST | DV478602 | Forward | 275 bp | 5’-ACCAACCTTGTGCCCTATCC (897–916) |
|  |  |  | Reverse |  | 5’-GGGCACCAGTCAACAAACTG (1171–1153) |
| *UBC18* | EST | DV481689 | Forward | 193 bp | 5’-GGAGGCACCTCAGGTCATTT (206-225) |
|  |  |  | Reverse |  | 5’-ATAGCGGTCATTGTCTTGCG (398-379) |
| *Ubi4* | EST | DV482834 | Forward | 126 bp | 5’-TGACACCATCGACAACGTGA (699-718) |
|  |  |  | Reverse |  | 5’-GAGGGTGGACTCCTTCTGGA (824-805) |
| *Ubi10* | EST | DV484269 | Forward | 237 bp | 5’-TCCACACTCCACTTGGTGCT (485-504) |
|  |  |  | Reverse |  | 5’-GAGGGTGGACTCCTTTTGGA (721-702) |
| *ARR4* | EST | DV482687 | Forward | 275 bp | 5’-TCCAGCTGCTCCTCAACAAC (94-113) |
|  |  |  | Reverse |  | 5’-CGTCAGGCATCTGTTGATCC (368-349) |
| *BAS1* | EST | DV486913 | Forward | 106 bp | 5’-ACGCTCGGCATGATCATAAA (125-144) |
|  |  |  | Reverse |  | 5’-GGATCATGCAACCGTCAGAG (230-211) |
| *CBF*3 | EST | DV485858 | Forward | 116 bp | 5’-TCGTCCTCCCTCACTGACAA (603-622) |
|  |  |  | Reverse |  | 5’-GCGTAGTAGAGGTCCCAGCC (718-699) |
| *Chitinase1* | EST | DV483341 | Forward | 100 bp | 5’-CAGTGGGGCTACTGCTTCAA (374-393) |
|  |  |  | Reverse |  | 5’-GCTGGTAGTTGGACTGCCCT (473-454) |
| *GA3OX2-1* | EST | DV489129 | Forward | 297 bp | 5’-TGTCCCTCGGGTACTTCCTC (76-95) |
|  |  |  | Reverse |  | 5’-CCCGATCGATCCCTAATAGC (372-353) |
| *HSC70* | EST | DV470232 | Forward | 209 bp | 5’-CAGGACTTCTTCAACGGCAA (1171–1190) |
|  |  |  | Reverse |  | 5’-ATGGTGGTGTTCCTTGGGAT (1379–1360) |
| *IAA1* | EST | DV479677 | Forward | 107 bp | 5’-CTACTGGGAACGAGGGGAAA (15-34) |
|  |  |  | Reverse |  | 5’-CCATGGGACATCTCCAACAA (121-102) |
| *NPR1* | EST | DV474769 | Forward | 137 bp | 5’-CTATCTGTCCAACGGCTCCA (429-448) |
|  |  |  | Reverse |  | 5’-TCACTACCTCCAGGCTGCTG (565-546) |
| *RD22* | EST | DV476780 | Forward | 110 bp | 5’-GTGACGTTACCGTGGCATTC (235-254) |
|  |  |  | Reverse |  | 5’-GCGTCGTCGTAGCAAGAGAG (344-325) |
| *GAPDH**  (for RT-PCR) | EST | DV482924 | Forward | 468 bp | 5’-ATGGGCAAGATTAAGATCGGAATCAACGG (78-106) |
|  |  |  | Reverse |  | 5’-AGTGGTGCAGCTAGCATTTGAGACAAT (545-519) |

The primers were designed based on the expression sequence tags (ESTs) deposited in the GeneBank using the Primer3 software (version 0.4.0) [24]. They had melting temperatures of a range of 50 - 60 ^o^C.

*This primer set was used only for RT-PCR analysis of the *GAPDH* expression. All other primers except for this were used for qRT-PCR reactions.
