# Supplementary material for: Exploring valid reference genes for gene expression studies in Brachypodium distachyon by real-time PCR
Source: BMC Plant Biol. 2008 Nov 7;8:112. doi: 10.1186/1471-2229-8-112 (PMC2588586; doi:10.1186/1471-2229-8-112)
Supplement: Additional file 2 — RT-PCR analyses of GAPDH transcript levels in different Brachypodium samples. Total RNA samples were isolated from plants grown under abiotic stresses (a), from different plant tissues (b), from plants treated with various growth hormones (c), or from plants at different developmental stages (d). The quality of the RNA samples was determined by electrophoretic analysis using the Labwork Image Acquisition and Analysis Program (Media Cybernetics, see Methods). [file 1471-2229-8-112-S2.ppt]

## Slide 1
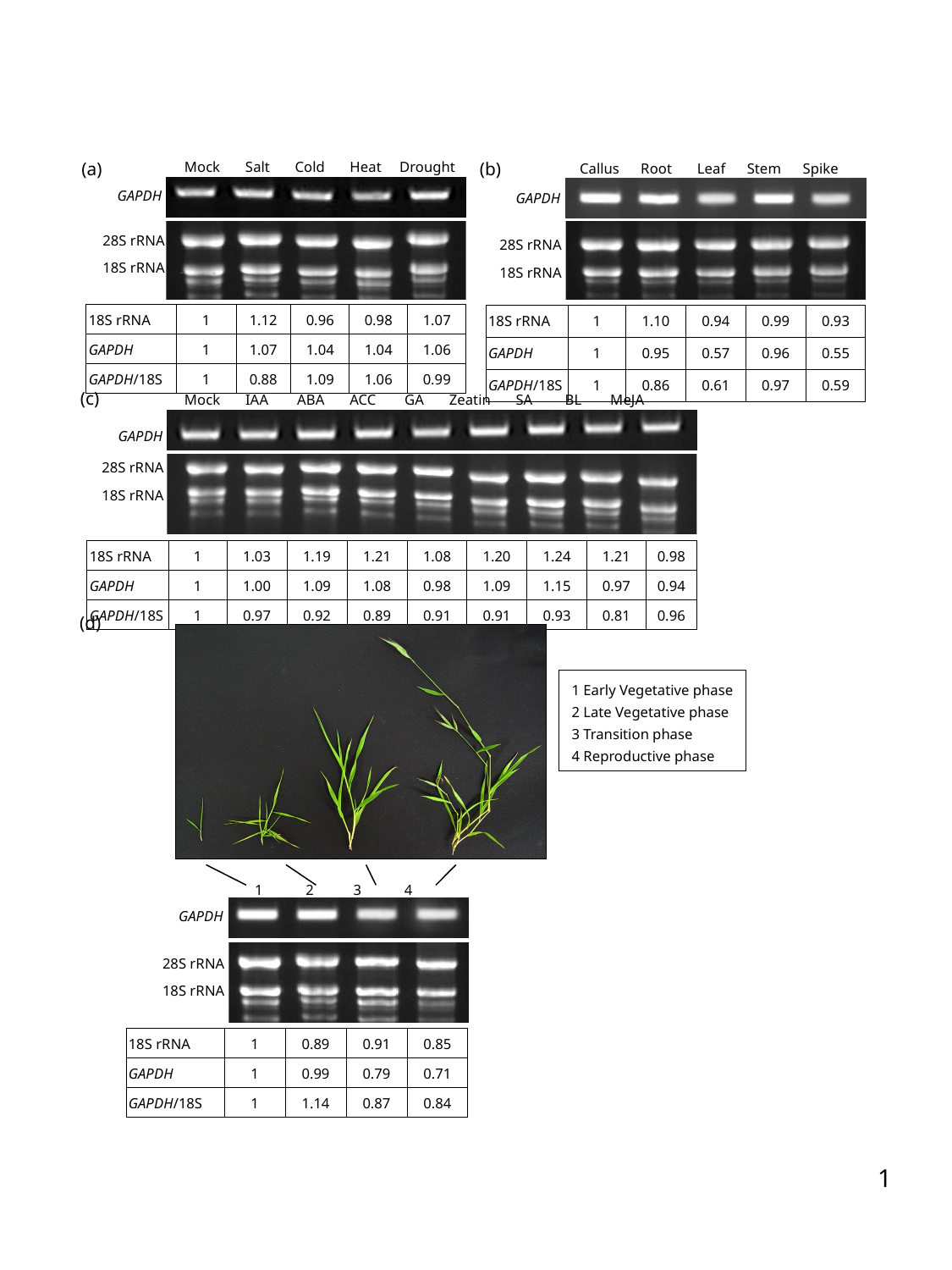

(a)
Mock Salt Cold Heat Drought
(b)
Callus Root Leaf Stem Spike
GAPDH
GAPDH
28S rRNA
28S rRNA
18S rRNA
18S rRNA
| 18S rRNA | 1 | 1.12 | 0.96 | 0.98 | 1.07 |
| --- | --- | --- | --- | --- | --- |
| GAPDH | 1 | 1.07 | 1.04 | 1.04 | 1.06 |
| GAPDH/18S | 1 | 0.88 | 1.09 | 1.06 | 0.99 |
| 18S rRNA | 1 | 1.10 | 0.94 | 0.99 | 0.93 |
| --- | --- | --- | --- | --- | --- |
| GAPDH | 1 | 0.95 | 0.57 | 0.96 | 0.55 |
| GAPDH/18S | 1 | 0.86 | 0.61 | 0.97 | 0.59 |
(c)
Mock IAA ABA ACC GA Zeatin SA BL MeJA
GAPDH
28S rRNA
18S rRNA
| 18S rRNA | 1 | 1.03 | 1.19 | 1.21 | 1.08 | 1.20 | 1.24 | 1.21 | 0.98 |
| --- | --- | --- | --- | --- | --- | --- | --- | --- | --- |
| GAPDH | 1 | 1.00 | 1.09 | 1.08 | 0.98 | 1.09 | 1.15 | 0.97 | 0.94 |
| GAPDH/18S | 1 | 0.97 | 0.92 | 0.89 | 0.91 | 0.91 | 0.93 | 0.81 | 0.96 |
(d)
1 Early Vegetative phase
2 Late Vegetative phase
3 Transition phase
4 Reproductive phase
1 2 3 4
GAPDH
28S rRNA
18S rRNA
| 18S rRNA | 1 | 0.89 | 0.91 | 0.85 |
| --- | --- | --- | --- | --- |
| GAPDH | 1 | 0.99 | 0.79 | 0.71 |
| GAPDH/18S | 1 | 1.14 | 0.87 | 0.84 |
1
