# Supplementary figures and images for: Exploring valid reference genes for gene expression studies in Brachypodium distachyon by real-time PCR
Source: BMC Plant Biol. 2008 Nov 7;8:112. doi: 10.1186/1471-2229-8-112 (PMC2588586; doi:10.1186/1471-2229-8-112)

## Slide 1
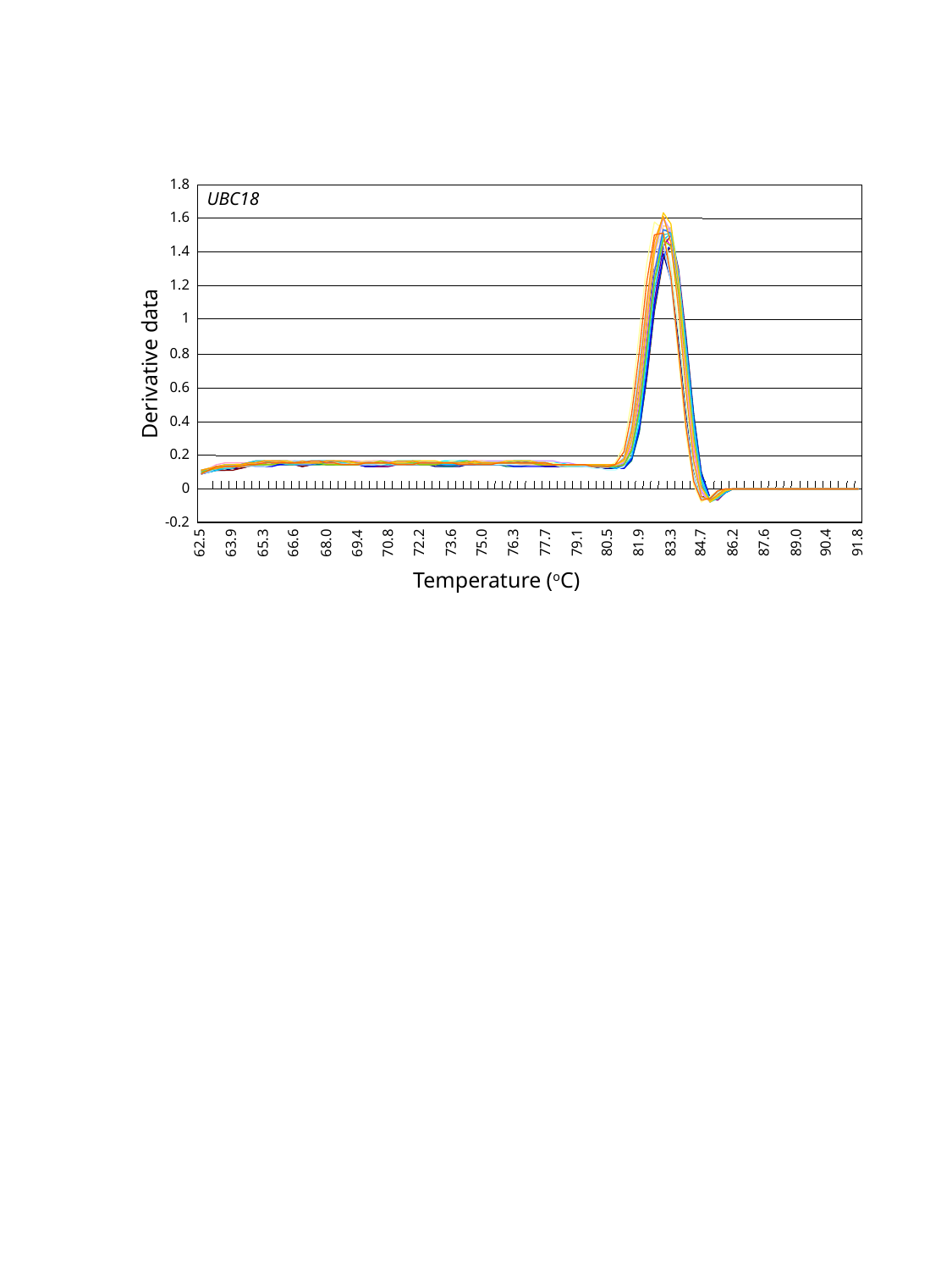

1.8
UBC18
1.6
1.4
1.2
1
0.8
Derivative data
0.6
0.4
0.2
0
-0.2
90.4
89.0
91.8
72.2
73.6
75.0
76.3
77.7
79.1
80.5
81.9
83.3
84.7
86.2
87.6
70.8
62.5
63.9
65.3
66.6
68.0
69.4
Temperature (oC)

Supplement: Additional file 3 — Dissociation curve data for UBC18 in growth hormone-treated samples. [file 1471-2229-8-112-S3.ppt]

## Slide 1
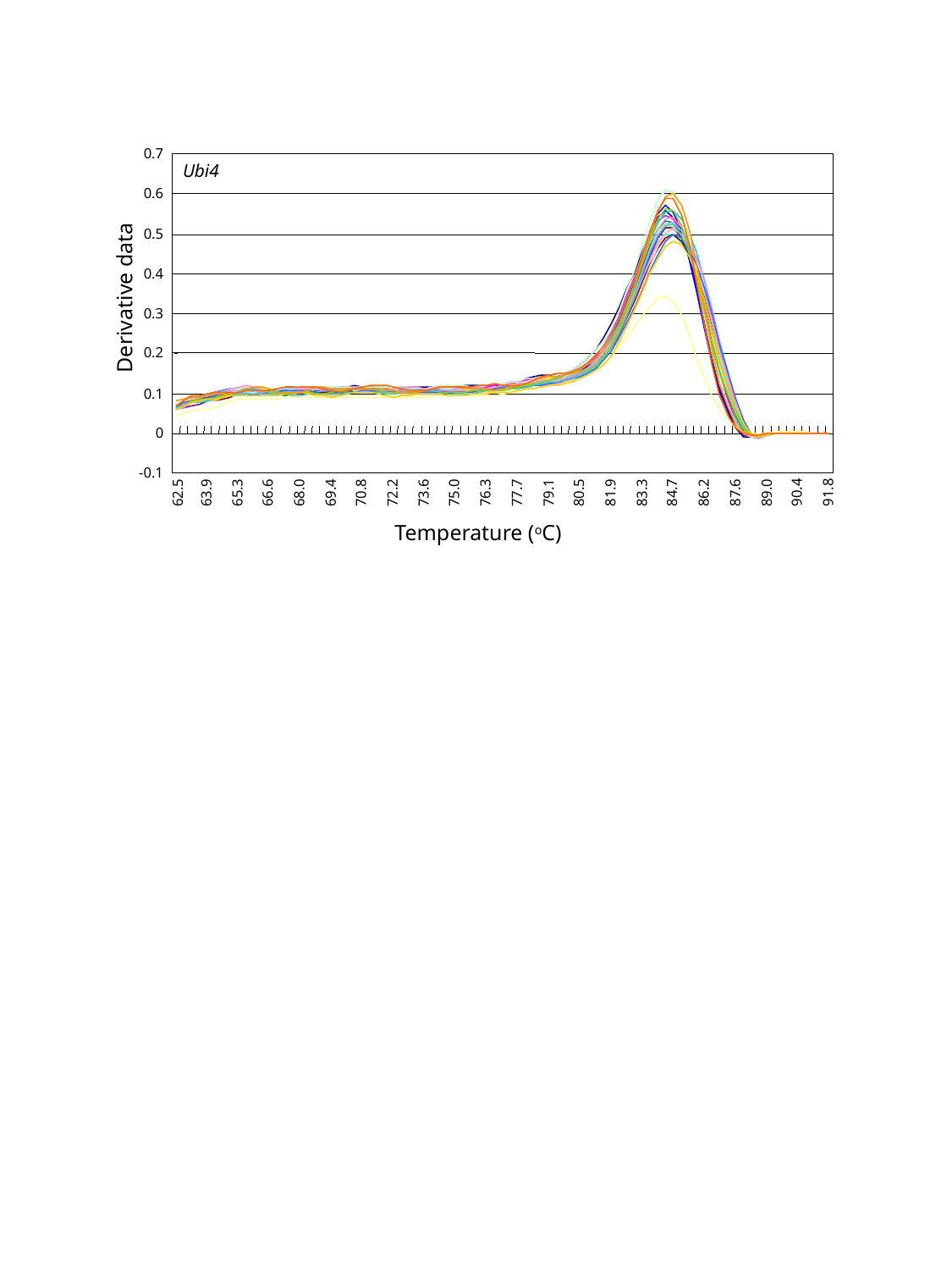

0.7
Ubi4
0.6
0.5
0.4
Derivative data
0.3
0.2
0.1
0
-0.1
90.4
91.8
62.5
63.9
65.3
66.6
68.0
69.4
70.8
72.2
73.6
75.0
76.3
77.7
79.1
80.5
81.9
83.3
84.7
86.2
87.6
89.0
Temperature (oC)

Supplement: Additional file 4 — Dissociation curve data for Ubi4 in growth hormone-treated samples. [file 1471-2229-8-112-S4.ppt]

## Slide 1
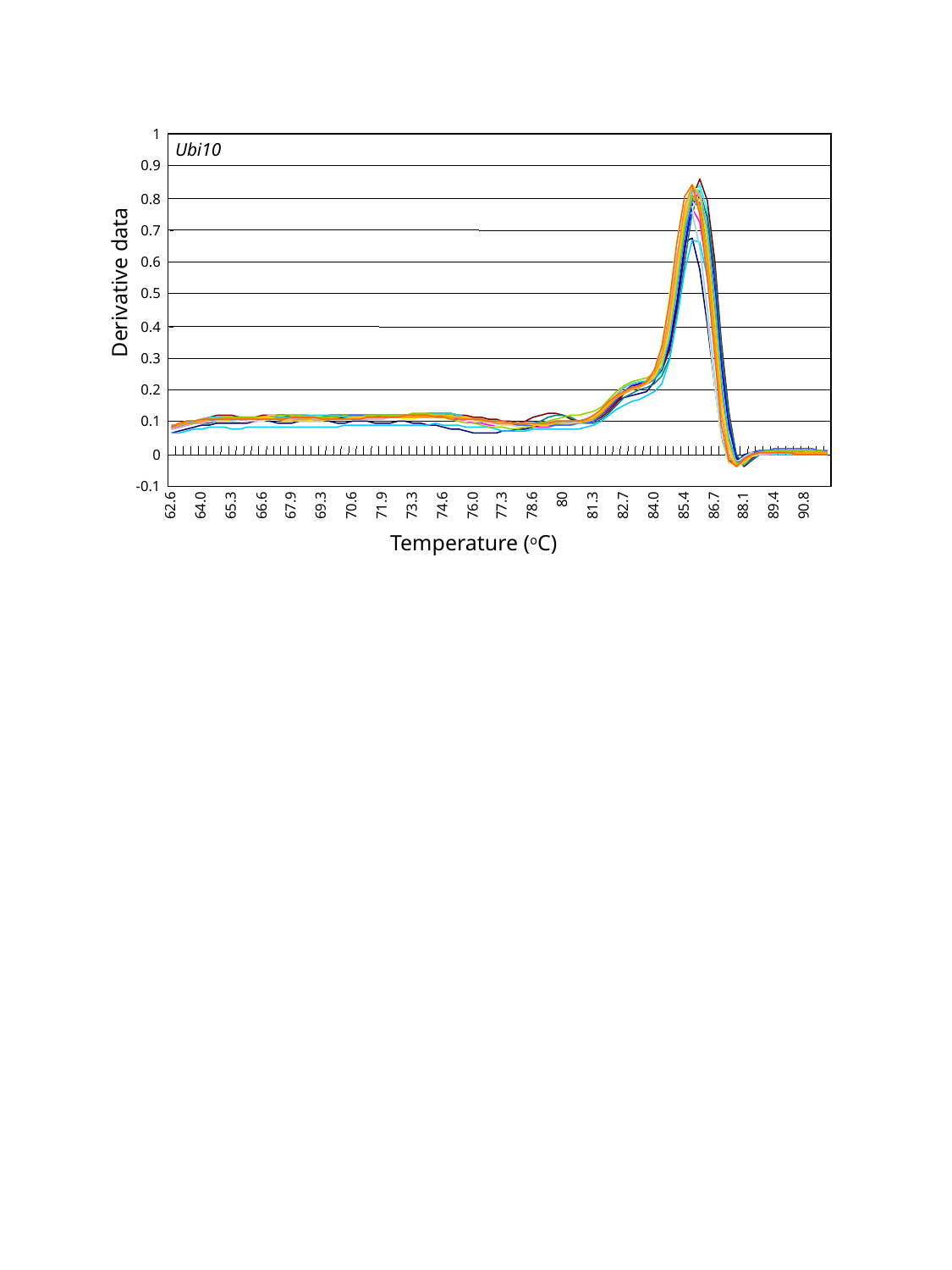

1
Ubi10
0.9
0.8
0.7
0.6
Derivative data
0.5
0.4
0.3
0.2
0.1
0
-0.1
80
62.6
64.0
65.3
66.6
67.9
69.3
70.6
71.9
73.3
74.6
76.0
77.3
78.6
81.3
82.7
84.0
85.4
86.7
88.1
89.4
90.8
Temperature (oC)

Supplement: Additional file 5 — Dissociation curve data for Ubi10 in growth hormone-treated samples. [file 1471-2229-8-112-S5.ppt]

## Slide 1
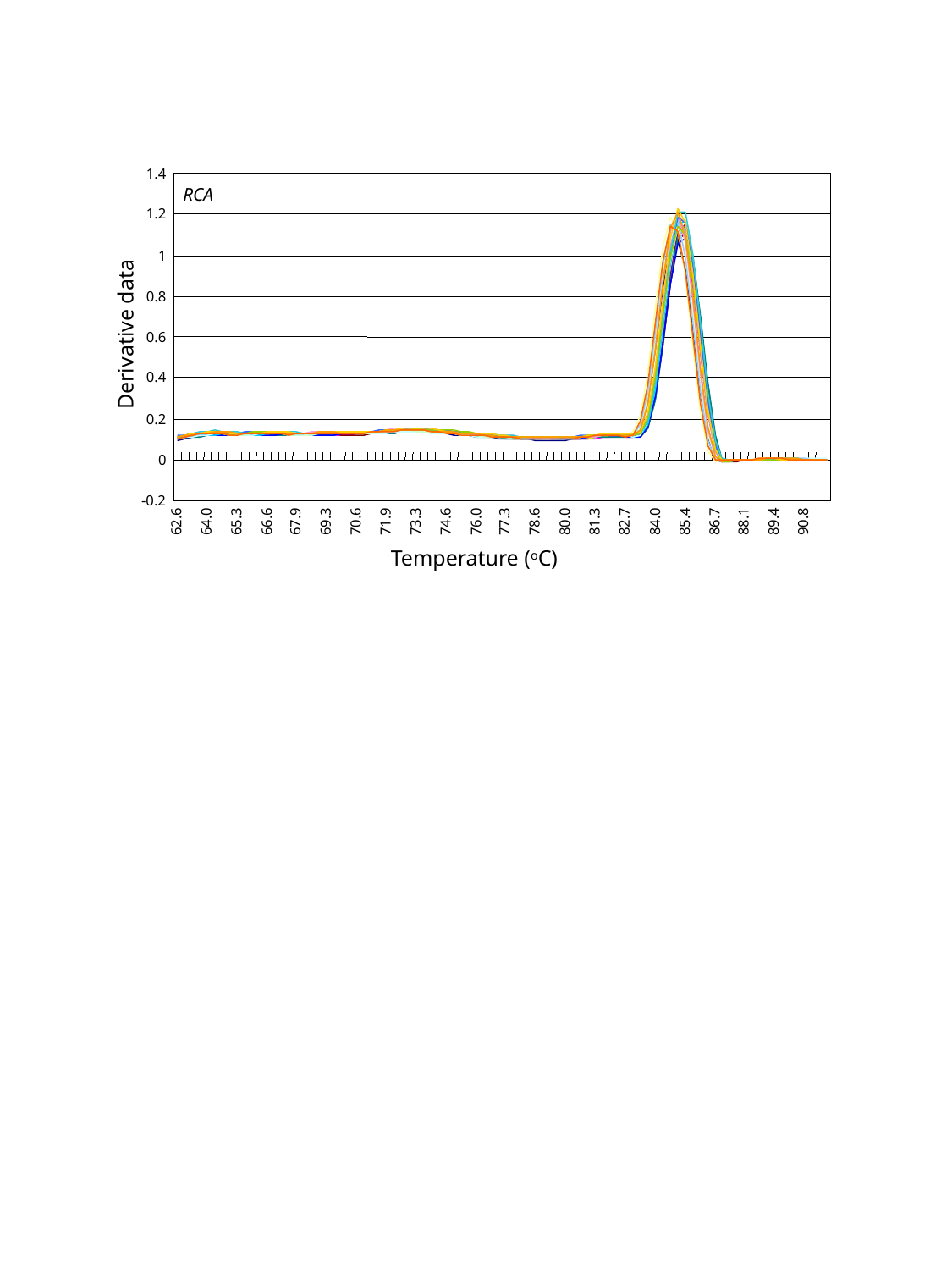

1.4
RCA
1.2
1
0.8
Derivative data
0.6
0.4
0.2
0
-0.2
80.0
62.6
64.0
65.3
66.6
67.9
69.3
70.6
71.9
73.3
74.6
76.0
77.3
78.6
81.3
82.7
84.0
85.4
86.7
88.1
89.4
90.8
Temperature (oC)

Supplement: Additional file 6 — Dissociation curve data for RCA in growth hormone-treated samples. [file 1471-2229-8-112-S6.ppt]

## Slide 1
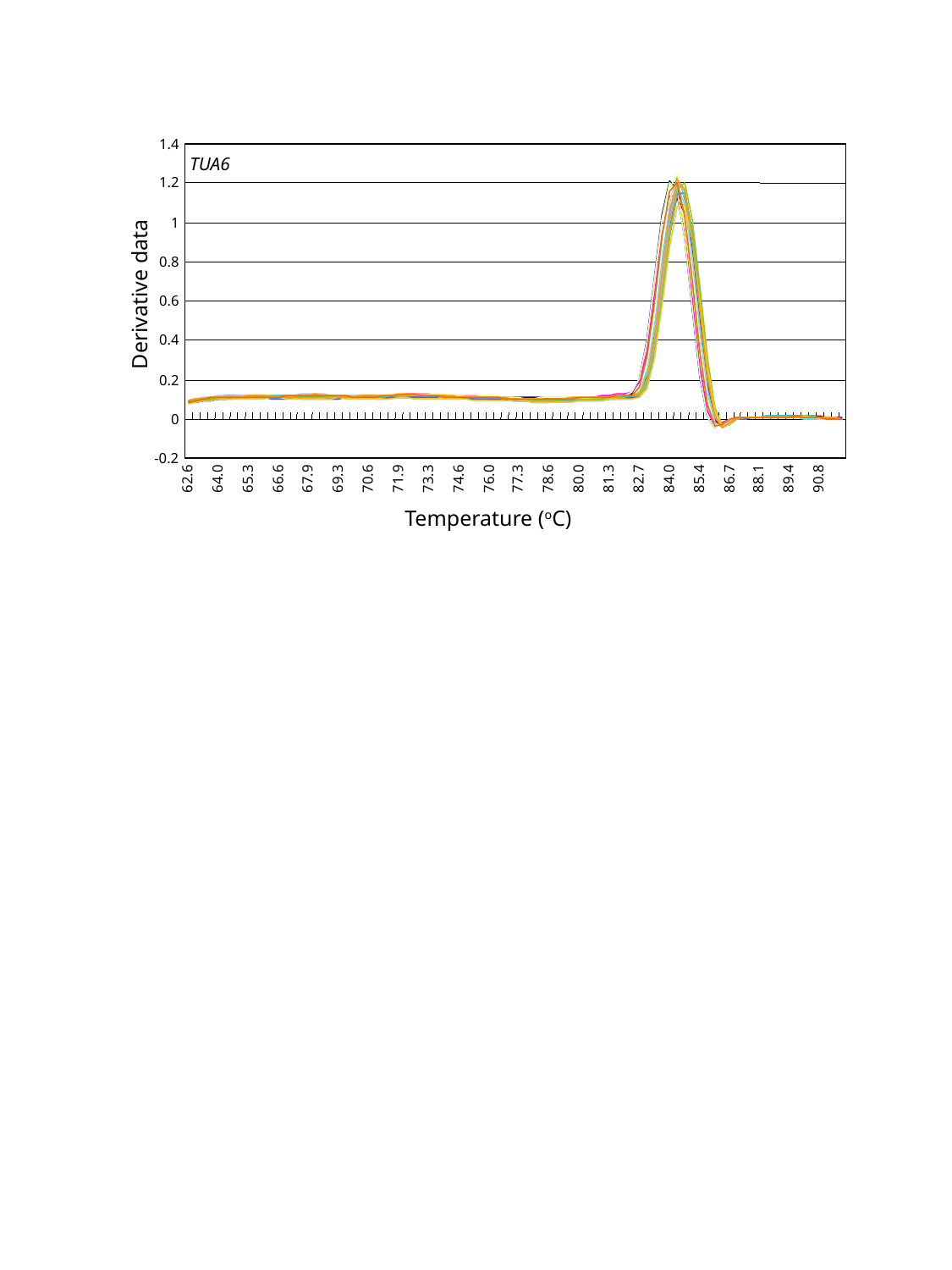

1.4
TUA6
1.2
1
0.8
Derivative data
0.6
0.4
0.2
0
-0.2
62.6
64.0
65.3
66.6
67.9
69.3
70.6
71.9
73.3
74.6
76.0
77.3
78.6
80.0
81.3
82.7
84.0
85.4
86.7
88.1
89.4
90.8
Temperature (oC)

Supplement: Additional file 7 — Dissociation curve data for TUA6 in growth hormone-treated samples. [file 1471-2229-8-112-S7.ppt]

## Slide 1
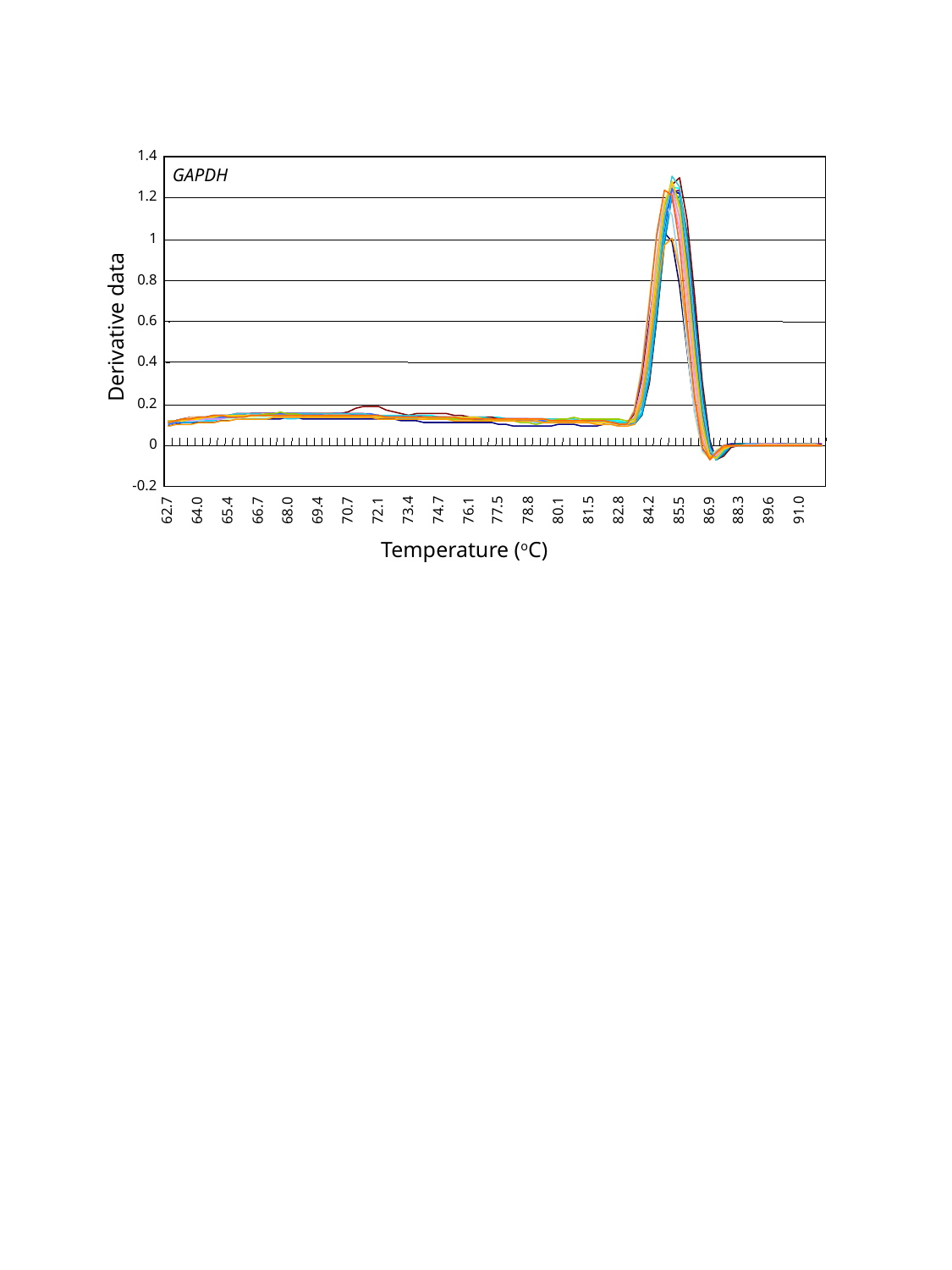

1.4
GAPDH
1.2
1
0.8
0.6
Derivative data
0.4
0.2
0
-0.2
73.4
74.7
76.1
77.5
78.8
80.1
81.5
82.8
84.2
85.5
86.9
88.3
89.6
91.0
62.7
64.0
65.4
66.7
68.0
69.4
70.7
72.1
Temperature (oC)

Supplement: Additional file 8 — Dissociation curve data for GAPDH in growth hormone-treated samples. [file 1471-2229-8-112-S8.ppt]

## Slide 1
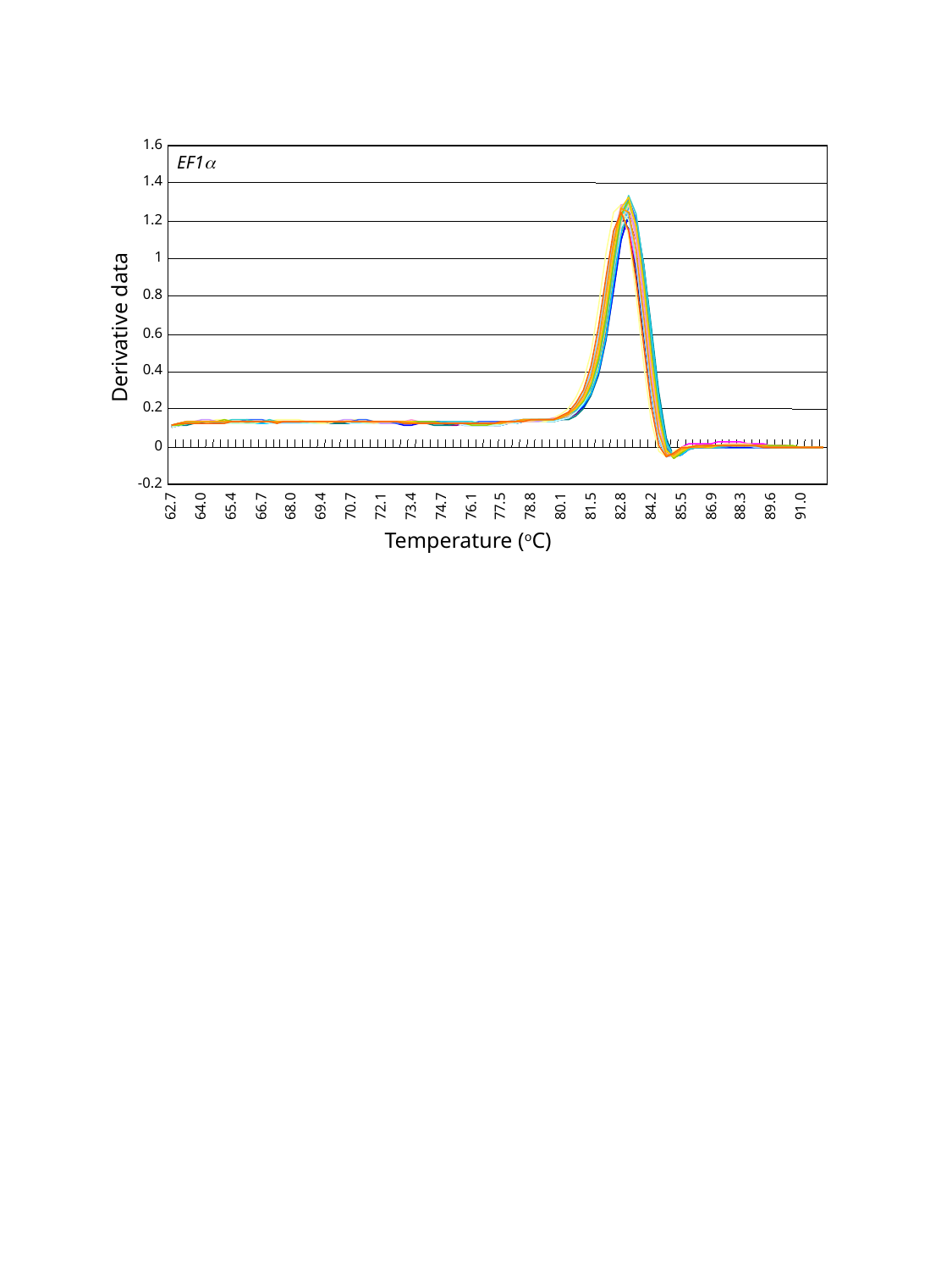

1.6
EF1α
1.4
1.2
1
0.8
Derivative data
0.6
0.4
0.2
0
-0.2
88.3
89.6
91.0
62.7
64.0
65.4
66.7
68.0
69.4
70.7
72.1
73.4
74.7
76.1
77.5
78.8
80.1
81.5
82.8
84.2
85.5
86.9
Temperature (oC)

Supplement: Additional file 9 — Dissociation curve data for EF1α in growth hormone-treated samples. [file 1471-2229-8-112-S9.ppt]

## Slide 1
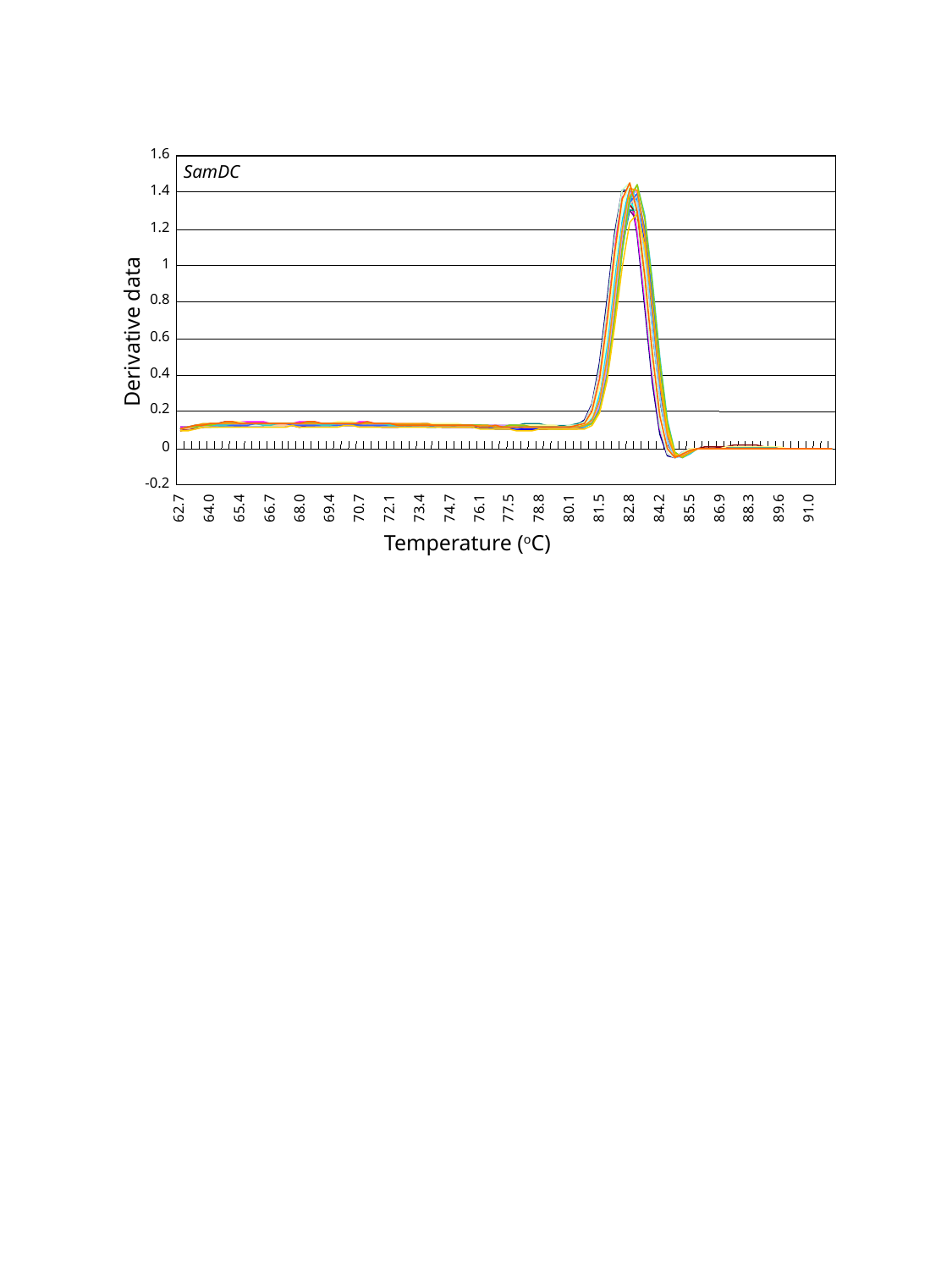

1.6
SamDC
1.4
1.2
1
0.8
Derivative data
0.6
0.4
0.2
0
-0.2
72.1
73.4
74.7
76.1
77.5
78.8
80.1
81.5
82.8
84.2
85.5
86.9
88.3
89.6
91.0
62.7
64.0
65.4
66.7
68.0
69.4
70.7
Temperature (oC)

Supplement: Additional file 10 — Dissociation curve data for SamDC in growth hormone-treated samples. [file 1471-2229-8-112-S10.ppt]

## Slide 1
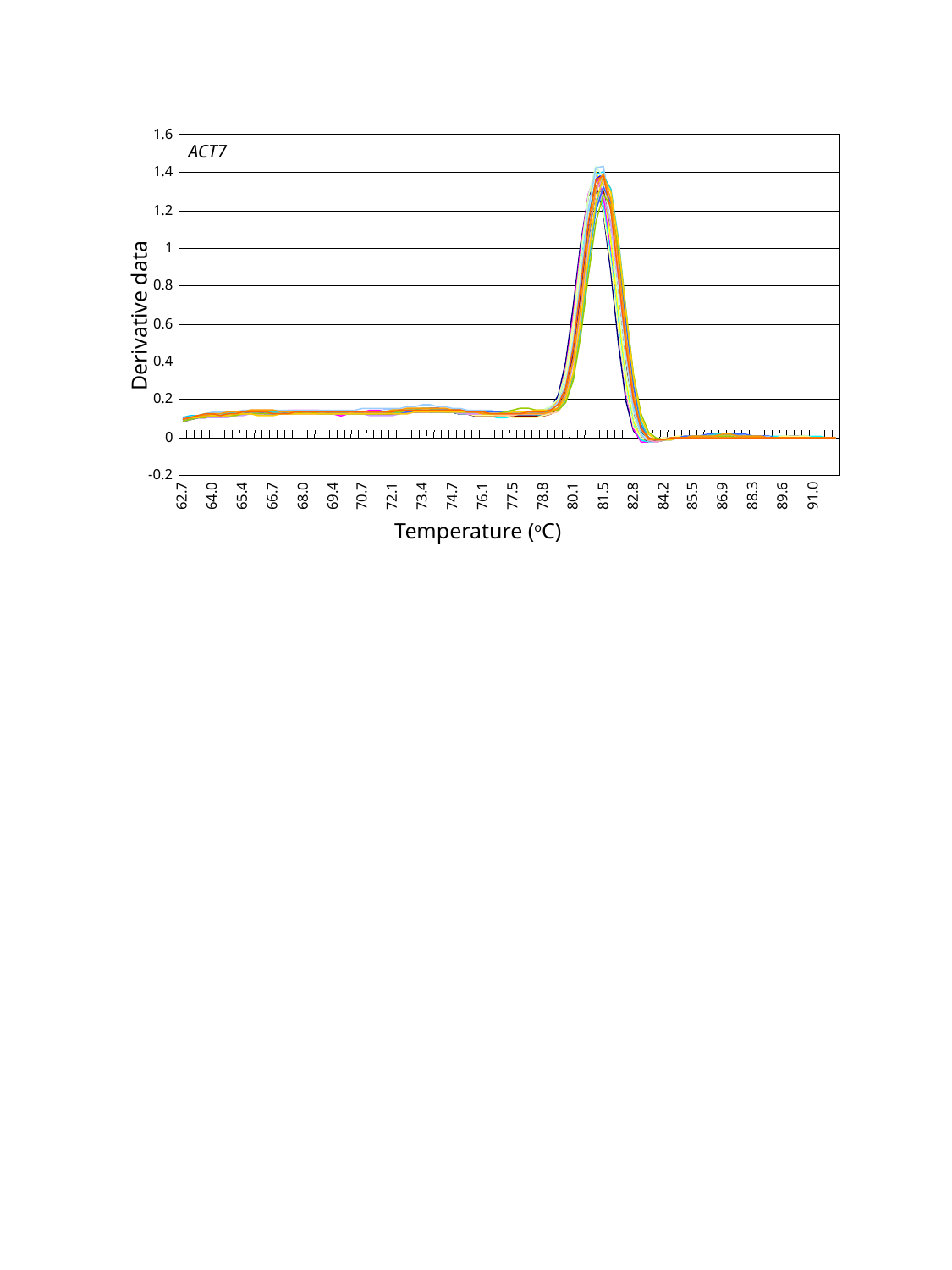

1.6
ACT7
1.4
1.2
1
0.8
Derivative data
0.6
0.4
0.2
0
-0.2
88.3
89.6
91.0
62.7
64.0
65.4
66.7
68.0
69.4
70.7
72.1
73.4
74.7
76.1
77.5
78.8
80.1
81.5
82.8
84.2
85.5
86.9
Temperature (oC)

Supplement: Additional file 11 — Dissociation curve data for ACT7 in growth hormone-treated samples. [file 1471-2229-8-112-S11.ppt]

## Slide 1
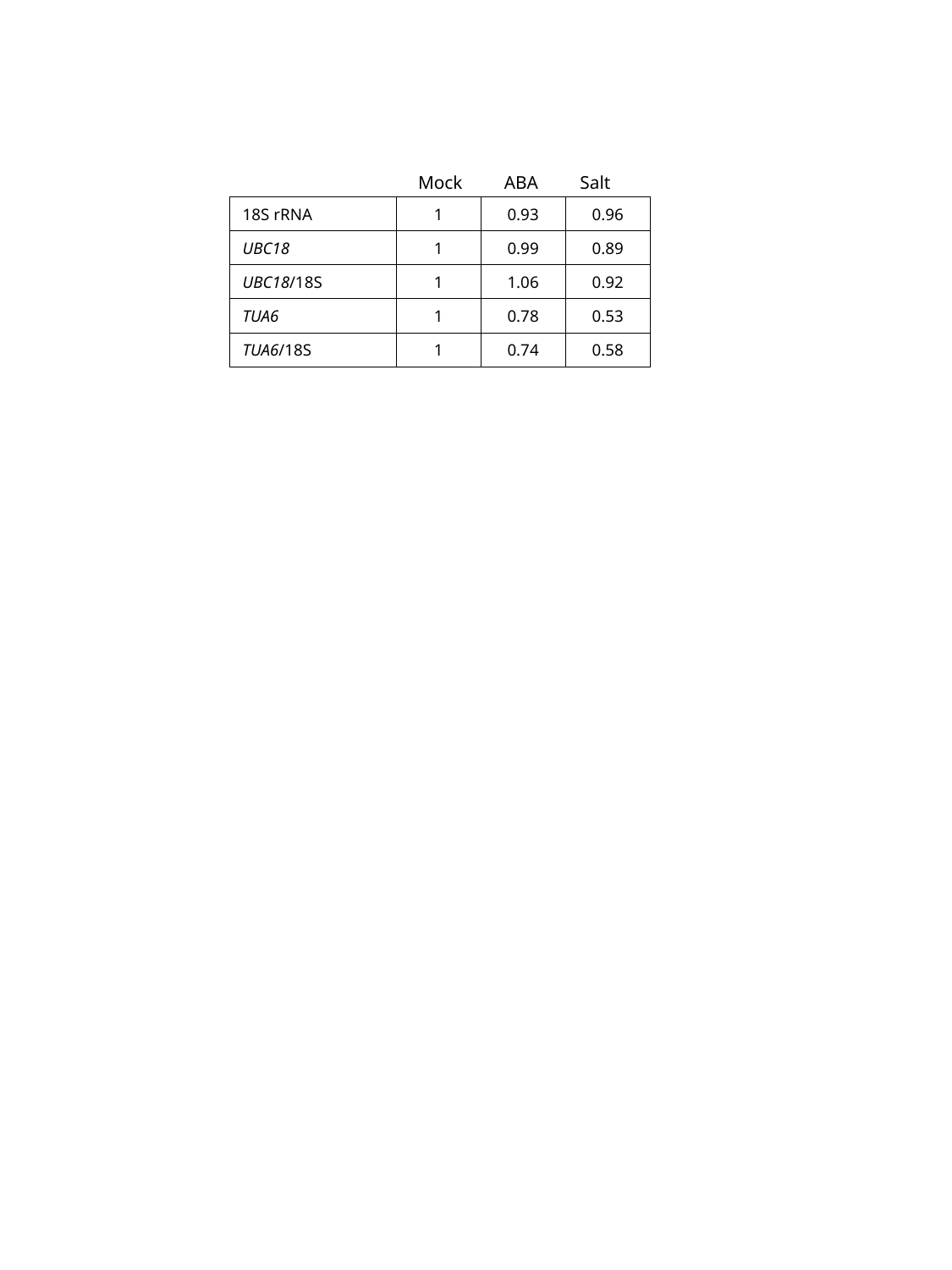

Mock ABA Salt
| 18S rRNA | 1 | 0.93 | 0.96 |
| --- | --- | --- | --- |
| UBC18 | 1 | 0.99 | 0.89 |
| UBC18/18S | 1 | 1.06 | 0.92 |
| TUA6 | 1 | 0.78 | 0.53 |
| TUA6/18S | 1 | 0.74 | 0.58 |

Supplement: Additional file 14 — Validation of the most suitable gene UBC18 using plants treated with ABA or high salt. RNA samples isolated from plants treated with ABA or high salt, as used in Additional file 13, were subject to RT-PCR of the UBC18 and TUA genes. The RT-PCR products were evaluated by electrophoretic analysis using the Labwork Image Acquisition and Analysis Program (Media Cybernetics). [file 1471-2229-8-112-S14.ppt]

## Slide 1
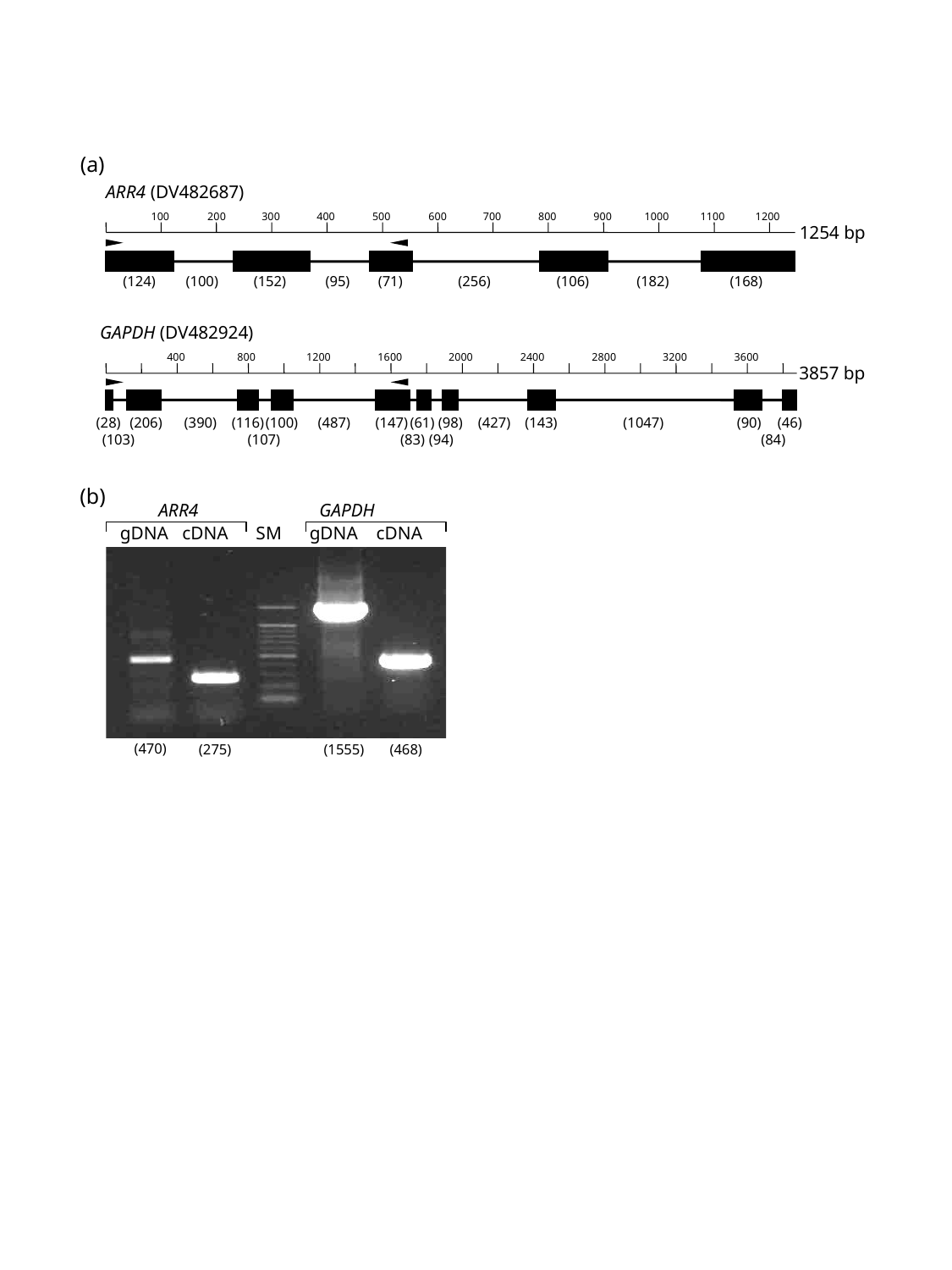

(a)
ARR4 (DV482687)
100
200
300
400
500
600
700
800
900
1000
1100
1200
1254 bp
(124)
(100)
(152)
(95)
(71)
(256)
(106)
(182)
(168)
GAPDH (DV482924)
400
800
1200
1600
2000
2400
2800
3200
3600
3857 bp
(28)
(206)
(390)
(116)
(100)
(487)
(147)
(61)
(98)
(427)
(143)
(1047)
(90)
(46)
(103)
(107)
(83)
(94)
(84)
(b)
ARR4 GAPDH
 gDNA cDNA SM gDNA cDNA
(470)
(275)
(1555)
(468)

Supplement: Additional file 15 — Genomic PCR and RT-PCR amplifications of the ARR4 and GAPDH genes. (a) The ARR4 and GAPDH gene structures. The gene sequences were extracted from the JGI 4X Brachy Sequence produced by the US Department of Energy Joint Genome Institute . The gene structures were predicted using the GENSCAN server . The black boxes denote exons. The numbers in parentheses indicate the sizes of exons and introns in base pairs (bp). The arrowheads mark the position and direction of the PCR primers. (b) Genomic PCR and RT-PCR. The PCR reactions were carried out for 30 cycles using either genomic DNA (gDNA) or primary cDNA (cDNA) synthesized from total RNA pretreated with a RNase-free DNase I. The PCR primers were those listed in Additional file 1. The numbers in parentheses indicate the sizes of PCR products in bp. The arrow marks a predicted PCR product. SM, size marker. [file 1471-2229-8-112-S15.ppt]
