# Supplementary material for: Exploring valid reference genes for gene expression studies in Brachypodium distachyon by real-time PCR
Source: BMC Plant Biol. 2008 Nov 7;8:112. doi: 10.1186/1471-2229-8-112 (PMC2588586; doi:10.1186/1471-2229-8-112)
Supplement: Additional file 12 — Brachypodium marker genes for gene expression studies in response to growth hormone treatments and abiotic stresses. The Brachypodium genes homologous to the Arabidopsis or rice genes that are regulated by growth hormones or abiotic stresses were examined by qRT-PCR. The sources of the gene accession numbers are those described in Table 1. [file 1471-2229-8-112-S12.doc]

**Additional file 14: Brachypodium marker genes for gene expression studies in response to growth hormone treatment**s and abiotic stresses

| Gene symbol | Response | Brachypodium | Rice | Arabidopsis |
| --- | --- | --- | --- | --- |
| *ARR4* | Cytokinin-inducible | DV482687 | LOC_Os02g35180 | AT1G10470 |
| *BAS1* | BR-inducible | DV486913 | LOC_Os02g11020 | AT2G26710 |
| *CBF3* | Cold-inducible | DV485858 | LOC_Os09g35030 | AT4G25490 |
| *Chitinase1* | ACC, JA-repressible | DV483341 | LOC_Os10g39680 | AT3G12500 |
| *GA3OX2-1* | GA-repressible | DV489129 | LOC_Os01g08220 | AT4G21690 |
| *HSC70* | Heat-inducible | DV470232 | LOC_Os03g60620 | AT5G02500 |
| *IAA1* | Auxin-inducible | DV479677 | LOC_Os03g53150 | AT3G04730 |
| *NPR1* | SA-inducible | DV474769 | LOC_Os01g56200 | AT5G45110 |
| *RD22* | ABA, high salt, drought-repressible | DV476780 | LOC_Os06g17000 | AT5G25610 |

The Brachypodium genes homologous to the Arabidopsis or rice genes that are regulated by growth hormones or abiotic stresses were examined by qRT-PCR. The sources of the gene accession numbers are those described in **Table 1**.
