# Supplementary material for: Exploring valid reference genes for gene expression studies in Brachypodium distachyon by real-time PCR
Source: BMC Plant Biol. 2008 Nov 7;8:112. doi: 10.1186/1471-2229-8-112 (PMC2588586; doi:10.1186/1471-2229-8-112)
Supplement: Additional file 13 — qRT-PCR data on Brachypodium genes regulated by growth hormones and abiotic stresses. Transcript levels were measured by qRT-PCR. Bars mark the standard error of the mean. The mean values were used as positive controls to determine the effects of growth hormone and abiotic stresses on reference genes. The gene accessions and the effects of each treatment on individual genes were summarized in Additional file 12. [file 1471-2229-8-112-S13.ppt]

## Slide 1
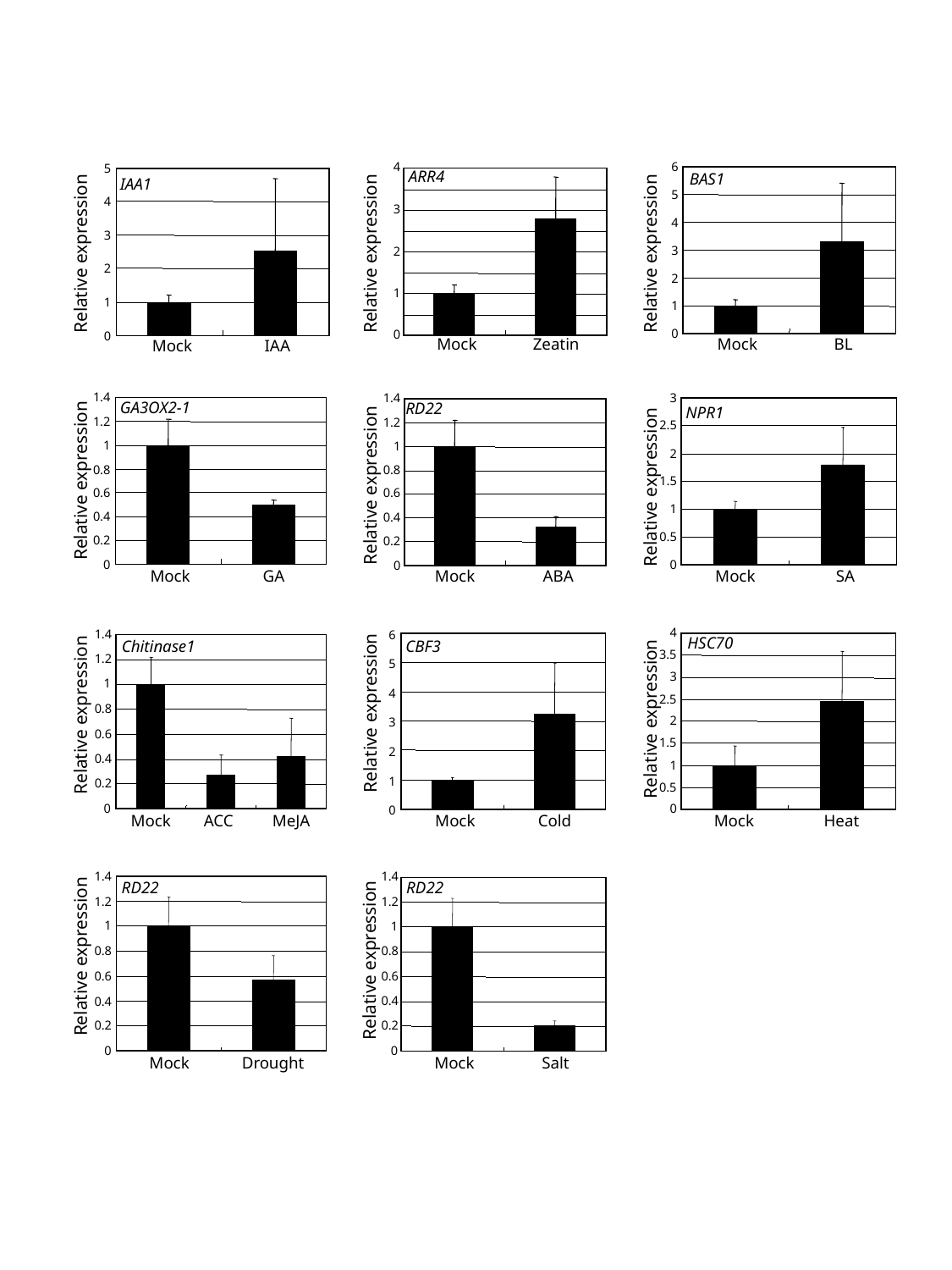

6
4
5
ARR4
BAS1
IAA1
5
4
3
4
3
3
Relative expression
Relative expression
Relative expression
2
2
2
1
1
1
0
0
0
Mock
Zeatin
Mock
BL
Mock
IAA
1.4
1.4
3
GA3OX2-1
RD22
NPR1
1.2
1.2
2.5
1
1
2
0.8
0.8
Relative expression
1.5
Relative expression
Relative expression
0.6
0.6
1
0.4
0.4
0.5
0.2
0.2
0
0
0
Mock
GA
Mock
ABA
Mock
SA
4
1.4
6
HSC70
Chitinase1
CBF3
3.5
1.2
5
3
1
4
2.5
0.8
Relative expression
Relative expression
Relative expression
2
3
0.6
1.5
2
0.4
1
1
0.2
0.5
0
0
0
Mock
ACC
MeJA
Mock
Cold
Mock
Heat
1.4
1.4
RD22
RD22
1.2
1.2
1
1
0.8
0.8
Relative expression
Relative expression
0.6
0.6
0.4
0.4
0.2
0.2
0
0
Mock
Drought
Mock
Salt
